# From Standard of Care to mRNA Cancer Vaccines and Spatial Architecture-Based Precision Therapy in PDAC: Challenges and Expectations

**DOI:** 10.3390/cancers18111824

**Published:** 2026-06-02

**Authors:** Elena X. Stea, Nikolaos Kydonakis, Dimitrios H. Roukos

**Affiliations:** 1 Centre for Biosystems and Genome Medicine, Ioannina University, 45110 Ioannina, Greece; e.stea@uoi.gr; 2 First Department of Surgery, Laiko General Hospital, National and Kapodistrian University of Athens, 11527 Athens, Greece; kydonakis_nikos@yahoo.gr

**Keywords:** pancreatic cancer, standard-of-care, mRNA cancer vaccine, comprehensive genomic profiling, molecular targeted therapy, ctDNA MRD, comprehensive TME analysis, spatial architecture, biomarkers, precision combination therapy

## Abstract

Pancreatic ductal adenocarcinoma (PDAC) is the most complex and aggressive disease with the worst rates of unresectable or metastatic disease at diagnosis, resistance to systemic therapy, and case fatality rate (CFR) among leading cancers. In non-metastatic disease, neoadjuvant treatment with modern chemotherapeutic regimens followed by surgical resection and/or adjuvant mFOLFIRINOX has significantly improved oncological outcomes. However, recurrence rates remain alarmingly high, while immune checkpoint inhibitors (ICIs) or molecularly targeted therapy have not yet demonstrated clinical benefits. Comprehensive genomic profiling through NGS-based approved assays such as TruSight Oncology 500 (TSO500) could guide targeted therapy. Rapidly evolving mRNA cancer vaccines and circulating tumor DNA (ctDNA)-based prediction of minimal residual disease (MRD) and recurrence risk hold great promise towards the realization of rational combination therapy to improve recurrence-free survival (RFS) and overall survival (OS). More recently, single-cell multiomics (SC MO), spatial proteomics and transcriptomics (SPT), artificial intelligence (AI), and systems biology have revolutionized cancer research, enabling holistic tumor microenvironment (TME) analysis. In this comprehensive review, we describe the latest advances and unmet needs in the standard of care of PDAC. Moreover, we discuss the expectations of ongoing randomized clinical trials of adjuvant mRNA vaccine-based therapy and ctDNA MRD testing as prognostic biomarkers, towards personalized treatment to improve RFS and OS in a medium-term perspective. With a longer perspective, we explore how harnessing SC MO, SPT, AI, and systems biology can reveal the 3D spatial organization of interacting cancer, immune, and stromal cells. Multi-dimensional TME-, TSO500- and ctDNA-based framework of dynamic biomarkers are of paramount importance to achieve an optimal patient-specific perioperative multimodal treatment combining precision immunotherapy, targeted drugs, and modern chemotherapy, translated into future practice-changing clinical trials, that could eliminate MRD towards recurrence prevention.

## Simple Summary

Pancreatic ductal adenocarcinoma (PDAC), with a 5-year overall survival of 13%, represents the challenge of the future. Despite surgical resection, the cornerstone of treatment, being feasible in only 20% of patients, with neoadjuvant and/or adjuvant modern chemotherapy, recurrence and mortality rates remain very high. Research efforts to combine chemotherapy with immune checkpoint inhibitors and targeted therapy have not yet translated into clinical practice. We describe the latest advances in the standard of care and the unmet needs to be overcome. Personalized mRNA cancer vaccines and circulating tumor DNA-based minimal residual disease detection to predict recurrence have rapidly translated into randomized clinical trials, holding promises to improve outcomes. Even greater expectations are provided by the unprecedented potential of innovative research integrating single-cell spatial multiomics, artificial intelligence, and systems biology to understand the extraordinarily complex and dynamically evolving PDAC tumor microenvironment. These advances constitute the hope of the future through the discovery of novel biomarkers guiding optimization of immune-based therapy, combinatorial treatment, tailored to individual patients.

## 1. Introduction

Evidence-based prevention screening for early detection and meaningful therapy in PDAC remains elusive. Most patients, ranging between 75–80% [1,2,3,4], are diagnosed with unresectable or metastatic (46%) disease with dismal prognosis. The 5-year overall survival (OS) is 13% [4], and the median survival is 4 months [5]. Moreover, disappointing are the estimates from the World Health Organization (WHO) for a considerable increase in incidence and mortality worldwide in 2040, including high-income countries [6]. Globally, PDAC presents the worst prognosis and case fatality rate (CFR) among major cancer types [6], considering the incidence and mortality. However, the positive message constitutes the recent remarkable 5-year OS increase from 32% in 2018 to 44% in 2023 for localized disease, which, however, accounts for 17% [4].

Although surgery is the cornerstone of treatment, recent advances in adjuvant 5-fluorouracil with leucovorin, irinotecan, and oxaliplatin (mFOLFIRINOX) [7] and, more recently, preoperative cisplatin, nab-paclitaxel, capecitabine, and gemcitabine (PAXG) [8], have demonstrated significant 5-year OS and 3-year event-free survival (EFS), respectively. However, recurrence rates remain alarmingly high, approximately 75%. The substantial intratumoural and intertumoural heterogeneity of PDAC [9] indicates the urgent need for the development of robust biomarkers and effective therapy for tailored treatment in selected patients to improve recurrence-free survival (RFS) and/or EFS.

In contrast to other major cancer types with successful combination therapy, in PDAC, targeted therapy remains challenging [10], while immunotherapy with immune checkpoint inhibitors (ICIs) has been disappointing [11]. Indeed, conventional tumor stage, clinicopathologic characteristics [12], single gene sequencing for the identification of germline mutations such as BRCA1/2 [13] and somatic mutations such as EGFR [14] and HER2 [15,16,17] have realized personalized treatment, thus having improved RFS, disease-free survival (DFS), and OS. Could PDAC-specific fundamental discoveries be translated into this combination therapy?

The rapidly evolving fields of adjuvant mRNA cancer vaccine in combination with the anti-PD-L1 antibody and mFOLFIRINOX [18], comprehensive genomic profiling (CGP)-based targeted therapy [19], as well as liquid biopsies for circulating tumor DNA (ctDNA)-based prediction of occult minimal residual disease (MRD) and recurrence [20], all pave the way towards rational combination therapy in PDAC. The importance of tumor microenvironment (TME) in tumorigenesis, tumor growth, metastatic dissemination, and response to therapy has previously been recognized in solid tumors [21,22]. Moreover, the recent dramatic advancements in understanding the extreme complexity, aggressiveness, and heterogeneous TME of PDAC encompassing cytotoxic T lymphocytes on one side and immunosuppressive TME on the other, through high-dimensional single-cell multiomics and spatial proteomics and transcriptomics, are shaping a novel roadmap towards personalization of immune-based combinatorial treatment [21,22].

In this comprehensive review, we describe the latest advances and unmet needs in the standard of care of PDAC, including screening, surveillance in high-risk individuals, lack of screening in asymptomatic adults, diagnosis, staging, and perioperative treatment in resectable and borderline resectable disease. We also address progress in understanding the capacity of primary tumor-specific cancer cells, such as heterogeneity, plasticity, and cancer stem cells (CSCs), for occult micrometastasis, resulting in MRD after treatment. Advances in ctDNA-based MRD and recurrence risk prediction have also been evaluated. Challenges and opportunities to achieve personalization of rational therapy combining molecular targeted therapy by overcoming KRAS being considered undruggable, personalized mRNA cancer vaccine coupled with anti-PD-L1 atezolizumab and mFOLFIRINOX, as well as prognostic and predictive biomarkers have also been explored. Ultimately, with a long-term perspective, we delve into the complexity and aggressiveness of the dynamically evolving PDAC TME by increasing integration of single-cell multiomics (e.g., genomics, epigenomics, transcriptomics, proteomics), spatial proteomics and transcriptomics, and artificial intelligence (AI)-based analysis of big clinical and cutting-edge research data. We discuss how advances in the innovative exploration of spatial organization of TME could be translated into novel biomarkers guiding personalization of precision multimodal therapy.

## 2. Epidemiology

The PDAC ranks 12th among all cancers globally and 6th in cumulative mortality [6]. PDAC is currently the 3rd and 4th leading cause of cancer-related mortality in the USA and Europe, respectively, and for 2040 it is projected to become the 2nd cause in the USA, while remaining in 4th place in Europe [6]. The stage distribution according to USA’s National Cancer Institute’s (NCI) Surveillance, Epidemiology, and End Results (SEER) program indicates that at diagnosis 17% of patients have localized disease (IA/IB) with 5-year OS 44%, 26% of patients have regional disease with a 5-year OS 17% and 46% of patients have metastatic disease with a 5-year OS 3% [4]. These data indicate how crucial the development of novel screening in asymptomatic adults is to improve early diagnosis and outcomes. The projection for 2040 suggests an increasing incidence and mortality due to the growing population, to around 9.2 billion people, and ageing worldwide. For instance, an estimated increase in incidence and mortality is 11% and 13% in Japan, respectively, 24% and 25% in Europe, 37% and 42% in the USA, as well as 58% and 64% in China (Table 1). The lowest risk of developing PDAC is in Africa (0.15%) and the highest in Western Europe (2.06%). The emerging challenge is the increase in global burden in adolescents and young adults, approximately 12% from 2022 to 2050 [23].

Based on the ratio between new cases (diagnoses) and deaths, the CFR should be considered when designing national health systems, fundamental and translational research strategies, as well as clinical trials towards improved outcomes in the future. Table 2 presents the CFR range in several very high human development index (HDI) countries and globally for five leading cancer types in 2022 and 2040. The PDAC currently has and will still in the future have the lowest CFR, followed by liver, lung, colorectal, and breast cancer in ascending order, according to the WHO estimates for new cases and deaths in 2022 and 2040 [6]. These results indicate the necessity for innovation in research and technologies as well as clinical trial design.

## 3. Tumorigenesis, Risk Factors, Spatiotemporal Evolution, Diagnosis

Understanding PDAC pathogenesis, including germline and somatic mutations as well as the impact of risk factors, spatiotemporal evolution until diagnosis, is crucial in the prevention, early detection, and successful treatment of PDAC. However, multiple challenges still persist.

### 3.1. Pathogenesis, Risk Factors, and Spatiotemporal Evolution

Somatic driver mutations contribute to tumorigenesis in 91% of patients, while germline mutations have been identified in the remaining 9% [24]. The most common driver mutations have been found in KRAS (88%), TP53 (61–74%), CDKN2A (16–44%), and SMAD4 (20–22%) [25]. PDAC can arise from precursor lesions, including pancreatic intraepithelial neoplasia (PanIN) in approximately 95%, intraductal papillary mucinous neoplasm (IPMN), and other less frequent precursor variants [26,27]. The KRAS protein has a critical role in metastasis and chemotherapy resistance [28].

Approximately two decades after the advent of Next Generation Sequencing (NGS) technologies [29], important advances have been noted in the understanding of tumorigenesis and dynamic evolution in solid tumors, including pancreatic cancer [30]. As part of the PCAWG of the ICGC and the TCGA, whole-genome sequencing (WGS) analysis by a single biopsy from bulk samples in 2658 cancers, including PDAC, timing analyses suggest that driver mutations often precede diagnosis by many years, if not decades.

More recently, advances in high-resolution single-cell sequencing and spatial transcriptomics technologies have increased our understanding of the heterogeneity and plasticity of PDAC. Enabling emerging predictive and multimodal therapeutic approaches by elucidating PDAC biology and response to therapy at the single-cell level [31].

Various lifestyle and inherited risk factors for pancreatic cancer are known. General risk factors for cancer include cigarette smoking and alcohol intake, while for pancreatic cancer, there are more specific risk factors such as diabetes and pancreatitis [10]. Heritability, including familial predisposition (with more than 1 first-degree relative) and germline mutations, ranges between 21–36% of patients with pancreatic cancer [32,33]. Individuals with germline mutations in BRCA1, BRCA2, PALB2, ATM, TP53, MLH1, MSH2, MSH6, and others account for approximately 10% of patients with PDAC [34] have a relative risk for developing pancreatic cancer ranging from 1.5 to 6.5 [35,36].

### 3.2. Screening and Surveillance

The US guidelines do not recommend screening in asymptomatic adults [37], while no biopsy or surgical resection is needed in most small cystic lesions. However, there is no suggestion for high-risk individuals with risk factors of age and lifestyle, nor are there any consensus guidelines of the International Cancer of the Pancreas Screening (CAPS) consortium. Screening for pancreatic cancer in asymptomatic adults is not recommended, given the low incidence and the lack of accurate screening modalities [38].

Annual contrast-enhanced magnetic resonance imaging (MRI)/magnetic resonance cholangiopancreatography (MRCP) and/or endoscopic ultrasound (EUS) is recommended in high-risk individuals with pathogenic germline mutations in genes such as CDKN2A, BRCA2, ATM, BRCA1, and others, as well as mutations in hereditary pancreatitis genes and a clinical phenotype consistent with hereditary pancreatitis [38].

Currently, surgery is recommended for IPMNs and PanINs at high risk for PDAC, such as a cyst diameter >4 cm or a pancreatic duct diameter (MPD) between 5 and 9.9 mm. However, given the high surgical risk for complications and late metabolic morbidity of pancreatoduodenectomy (PD), duodenum-preserving pancreatic head resection (DPPHR) for IPMN is proposed [39].

### 3.3. Diagnosis and Staging

In individuals with clinical symptoms and suspicion of PDAC, the imaging modality recommended is contrast-enhanced three-phase computed tomography (CT). The differentiation between PDAC and other periampullary carcinomas can be challenging. MRI or endoscopic ultrasound (EUS) can be performed in individuals with a contraindication for contrast-enhanced CT. Beyond CT or MRI, positron emission tomography (PET)/CT or PET/MRI scan can also be considered in specific cases [38,40]. In patients with non-metastatic disease and given the current trend towards preoperative (neoadjuvant) chemotherapy, not only in borderline resectable, but also in resectable tumors [8], as well as initiation of chemotherapy in locally advanced disease, EUS-guided fine needle biopsy (FNB) or aspiration (FNA) is recommended [38,40].

In a relatively large-scale observational study 1.835 non-metastatic PDAC patients at diagnosis were included and classified as potentially resectable in 346 (18.9%), borderline resectable (BR) in 531 (28.9%), and locally advanced (LA) in 958 (52.2%). Resection was performed in 697 patients (38%). Independent prognostic factors for OS at diagnosis were tumor anatomy (A: BR and LA disease), biological factors (B: CA 19-9 > 500 U/mL), and conditional (C: WHO performance status ≥ 1). Staging of patients with non-metastatic disease at diagnosis should be based on ABC factors [3].

## 4. Treatment: Advances and Challenges

### 4.1. Perioperative Treatment

Following staging, as mentioned above, decision making for surgery with perioperative treatment in non-metastatic resectable, borderline resectable, and locally advanced disease, while systemic therapy or palliative care in metastatic disease is recommended. Surgical resection remains the cornerstone of curative-intent treatment. The aim of surgery for PDAC is the complete surgical (R0) resection and appropriate lymph node dissection to prevent residual cancer cells at the resection margins and/or undissected lymph nodes leading to locoregional recurrence, considering the intrinsic and acquired resistance to standard chemotherapy. Recent data have established the non-inferiority of minimally invasive pancreatectomy regarding radical resection rates, when performed in high-volume centres [41,42]. However, despite advances in surgical resection, recurrence and death rates were alarmingly high. Therefore, perioperative chemotherapy with or without radiotherapy in the adjuvant and neoadjuvant setting has been evaluated to improve the poor oncological outcomes.

#### 4.1.1. Adjuvant Therapy

Several phase III randomized clinical trials (RCTs) have previously demonstrated the efficacy of gemcitabine or chemoradiation to improve survival [43,44]. Subsequently, the phase III RCT ESPAC4 trial has demonstrated that mOS was significantly longer in the gemcitabine plus capecitabine group as compared to the gemcitabine group [45]. However, the 5-year RFS was 18.6%. Recently, the results of the phase III PRODIGE24/CCTGPA.6 (NCT01526135) RCT have demonstrated significantly improved 5-year DFS in favor of the chemotherapeutic regimen mFOLFIRINOX versus gemcitabine monotherapy [26.1% versus 19.0% respectively ([HR], 0.66; 95% CI, 0.54–0.82; *p* < 0.001)] and 5-year OS [43.2% versus 31.4% respectively (HR, 0.64; 95% CI, 0.52–0.80; *p*  <  0.001)] [7]. On the basis of these findings, the adjuvant mFOLFIRINOX is the standard of care in resectable PDAC. However, the incomplete resection (R1) with positive resection margins was very high (64.6%) in the mFOLFIRINOX group.

#### 4.1.2. Neoadjuvant Treatment

Evidence indicates that neoadjuvant treatment, including conventional chemotherapy, radiotherapy, molecular-targeted therapy, as well as immunotherapy and particularly ICIs, as compared to upfront surgery and adjuvant therapy in many major cancer types, such as lung, breast, and rectal tumors, provides a series of advantages: increased resectability, R0 resection, and improved RFS and OS [46,47]. Neoadjuvant cancer therapy before surgery presents an optimal platform for breakthrough research and clinical applications. Moreover, rapid assessment of pathological responses such as complete (pCR), major (MPR), partial (pPR), or non-response (pNR) enables decision-making on rational adjuvant treatment [47].

Based on the advantages of pre-operative treatment, current research focuses on the development of effective neoadjuvant treatment followed by surgery and adjuvant chemotherapy for the treatment of PDAC. Table 3 summarizes the results from neoadjuvant phase II and III clinical trials. Based on promising results from phase II trials and observational studies using mFOLFIRINOX, FOLFOX, gemcitabine/nab-paclitaxel, and/or S-1 [48,49,50,51,52,53,54,55,56,57,58,59,60,61], phase III RCTs have been conducted [8,62]. More recently, the phase III randomized Cassandra trial has evaluated the superiority of preoperative PAXG over mFOLFIRINOX in patients with stage I–III resectable and borderline resectable PDAC [8]. In a total of 260 eligible patients, 132 were assigned to PAXG and 128 to mFOLFIRINOX. After a median follow-up of 28.5 months, the median EFS was significantly longer in the PAXG arm (16 months) over the mFOLFIRINOX arm (10.2 months) [HR 0.63, 95% CI 0.47–0.84]. The 3-year EFS rate was 40% in the PAXG group, compared to 15% in the mFOLFIRINOX group. Based on these findings, the authors concluded that neoadjuvant PAXG could be considered a standard option for resectable or borderline resectable PDAC [8]. Despite the low EFS rate in the mFOLFIRINOX group, further clinical trials are needed to determine whether mFOLFIRINOX is inappropriate in the neoadjuvant setting, considering that in the adjuvant setting, mFOLFIRINOX had 26% 5-year DFS [7]. Future trials evaluating the clinical utility separately of resectable or borderline resectable with primary endpoints EFS, RFS, cancer-specific survival, and OS will determine the standard therapy of PDAC.

Although high-level evidence from phase III RCTs is still lacking, most oncologists and surgeons from tertiary institutions prefer neoadjuvant therapy for patients with borderline resectable disease [38]. Three ongoing phase III RCTs are testing the clinical benefit of neoadjuvant treatment followed by surgery and adjuvant mFOLFIRINOX (perioperative management) as compared to standard adjuvant mFOLFIRINOX (12 cycles) after surgery in resectable PDAC: PREOPANC-3 (NCT04927780), Alliance A021806 (NCT04340141), and NeoFOL-R (NCT05529940). All these trials use the regimen mFOLFIRNOX either with 8 cycles before and 4 cycles after surgery or with 6 cycles before and 6 cycles after surgery. The primary endpoint in all these studies is OS, whereas secondary endpoints include DFS, distant metastasis-free survival (DMFS), locoregional progression-free survival (PFS), time to locoregional recurrence, time to distant metastases, R0 rate, and adverse effects and pCR. Despite the positive results of the CASSANDRA trial in not only borderline resectable, but also resectable disease [8], definitive conclusions on the clinical benefits of preoperative treatment in resectable disease should await the completion of these ongoing phase III RCTs. Until then, the European Society for Medical Oncology (ESMO) Guidelines do not recommend neoadjuvant therapy, outside of clinical trials in resectable PDAC [40].

### 4.2. Unmet Needs

Despite the latest advances in neoadjuvant chemotherapy, surgery, and adjuvant chemotherapy with the most recent regimens such as mFOLFIRINOX and PAXG in phase III RCTs, recurrence rates in resectable and borderline resectable remain alarmingly high [7,8]. Overall recurrence ranges between 67–85% [7,63,64]. DFS and OS depend on tumor stage [I–III], classification in resectable or borderline resectable, and R0 versus R1 resection. Overall survival in the USA ranges between 44% in localized, 17% locoregional, and 3% in metastatic disease [4]. Given that recurrence originates from MRD, these data indicate the intrinsic and acquired resistance to current chemotherapeutic regimens. Therefore, current research focuses on early cancer detection, MRD detection, and the development of more effective perioperative combination therapies such as molecular targeted therapy and immunotherapy, considering the successful integration of these therapies in lung cancer, melanoma, and triple-negative breast cancer [65,66,67,68]. Considering screening and surveillance in high-risk adults with germline mutations in specific genes, EUS-guided FNB is required for pathological diagnosis prior to neoadjuvant treatment, and the recent trend in perioperative therapy, multidisciplinary consultation of experts in high-volume centers is recommended [38].

## 5. Detection of MRD: Challenges and Opportunities

MRD or residual cancer cells or persister cancer cells following current standard treatment with curative intent in solid tumors represent a major cause of overt recurrence and mortality [69,70]. Occult micrometastasis at diagnosis leads to micrometastatic residual disease responsible for subsequent recurrence in solid tumors, including PDAC [71,72,73]. Despite complete surgical (R0) resection of the primary tumor, adequate lymph node dissection, and perioperative standard multimodal therapy, recurrence rates remain alarmingly high in some tumors, particularly in PDAC. Detection, characterization, and meaningful therapy of residual cancer cells to prevent recurrence remain an unmet challenge [74]. Over the past decade, intensive breakthrough research has been focused on pre-clinical studies and liquid biopsies, particularly ctDNA detection, to predict MRD and recurrence risk.

### 5.1. Experimental Characterization of MRD: Direct Evidence in Pre-Clinical Studies

Given the invisibility of MRD not only by imaging-based technologies, such as CT, MRI, and PET scans, but also by molecular and precision imaging [10,75], emerging investigations are exploring MRD through preclinical models [76,77].

The CSCs concept was introduced approximately 5 decades ago, as a minor cell subpopulation of intratumor heterogeneity (ITH) [78,79]. Based on the potentially important clinical implications of this CSC theory regarding tumorigenesis, relapse, and metastasis, the model has been extensively investigated over the past decades [80,81].

Several models have been proposed to elucidate the micrometastatic residual disease following complete surgical resection of the primary tumor in colorectal cancer (CRC). Pioneering studies have uncovered the following: 1. LGR5+ cancer stem cells are responsible for primary CRC growth, yet are necessary for metastasis formation in experimental models [82], 2. Disseminated differentiated tumor cells initiate metastases and, through plasticity, produce LGR5+ cancer stem cells upon reaching the liver [83] and 3. A recent study based on in vitro and in vivo data indicates that both CSC and non-CSC have the capacity to form micrometastases due to cellular heterogeneity [84]. 4. Lastly, a most recent landmark study integrating single-cell transcriptome analysis in samples from patients with resectable CRC has demonstrated that metastatic recurrence following surgical resection of primary CRC arises from residual high-relapse cells (HRC) sub-population that are neither differentiated nor stem-like in a humanized patient-derived mouse model [85]. However, current research on CSCs and HRCs relies heavily on mice and other preclinical models, which cannot fully replicate the human tumor microenvironment, thus providing limitations to successfully be translated into clinical trials [86].

### 5.2. Indirect Evidence: ctDNA MRD

The unique capacity of longitudinal ctDNA analysis as a prognostic biomarker to detect MRD in both perioperative settings and patient monitoring has transformed cancer research towards therapeutic decisions and recurrence prevention [87]. Indeed, the clinical use of ctDNA MRD as a dynamic biomarker reflecting cancer dynamics, spatiotemporal tumor evolution, interconnectedness between cancer cells and immune systems within the TME, justifies the current explosion in translational and clinical research in the development and clinical validation of ctDNA academic and commercial assays [30,88,89]. However, multiple significant challenges remain to detect MRD with high sensitivity and specificity rates, which are crucial for clinical implementation. These hurdles include low concentration of tumor DNA in the plasma, thus requiring high-depth assays to detect variant allele frequencies (VAFs) as low as 0.01% [90,91].

**Figure 1:**
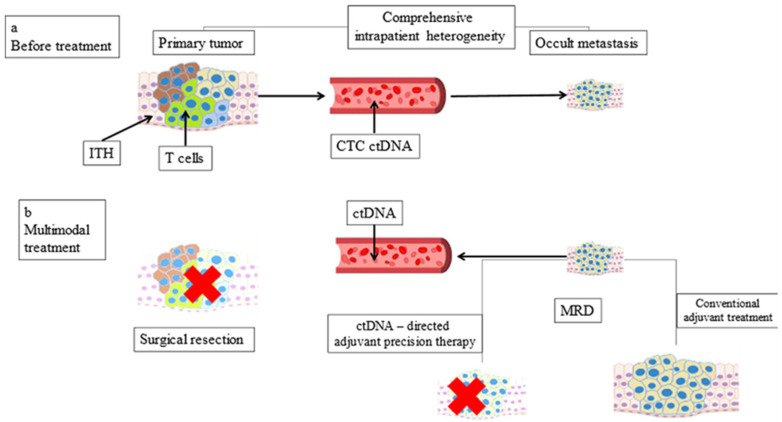
Serial profiling of circulating tumor DNA (ctDNA) to detect and treat minimal residual disease (MRD) over the disease course in early-stage patients. (**a**). Intrapatient heterogeneity. Intrapatient heterogeneity encompasses the primary tumor and ctDNA variability as well as occult micrometastatic disease in some patients treated with curative intent. Primary intratumor heterogeneity of both cancer and immune cells, as well as their interactions, represents a dynamically evolving tumor ecosystem. (**b**). Emerging ctDNA MRD-based multimodal treatment. Following complete surgical resection (R0), a reliable ctDNA-based detection indicates the presence of MRD. The analytical and clinical validity of diverse ctDNA MRD assays with high sensitivity in predicting personalized recurrence risk can guide personalization of combination therapy to prevent recurrence [73]. Abbreviations: ITH, intratumor heterogeneity; CTC, circulating tumor cells; ctDNA, circulating tumor DNA; MRD, minimal residual disease.

The presence of circulating tumor cells (CTCs), released from the primary tumor into circulation, is responsible for colonization in the distant organs, forming micrometastases and subsequently leading to micrometastatic residual disease after surgery with curative intent and recurrences (Figure 1 [73]). This occult residual disease cannot be detected with imaging scans or serum biomarkers, underscoring the clinical importance of ctDNA assays. Rapid advances over the past decade in the development and validation of ctDNA-based MRD detection, referred to as ctDNA MRD [87], are based on two methodological strategies: a. tumor-agnostic or tumor-naïve approaches and b. tumor-informed strategies. Tumor-informed strategies are based on tissue sequencing to identify tumor-specific mutations to guide personalized treatment and patient monitoring. Second, tumor-agnostic, or tumor-naïve or ctDNA-only approaches, enable the detection of genomic or epigenomic alterations through predefined panels directly from plasma without prior tissue analysis.

The detection of common cancer-associated genomic or epigenomic alterations utilizes predefined panels to detect common cancer-associated genomic or epigenomic alterations directly from plasma without prior tissue analysis, and identify tumor-specific mutations through initial tissue sequencing to guide ctDNA monitoring [92,93,94]. This unprecedented potential to detect MRD or residual cancer cells or persistent cancer cells after perioperative treatment has led to a plethora of ongoing clinical studies and assays in solid tumors [73]. Particularly in colorectal, lung, breast, and gastric cancer, rapid advances have reached phase II and III clinical trials towards the development of meaningful therapy to eliminate MRD in ctDNA-positive patients with minimal disease burden [95,96,97]. Multiple studies have evaluated the analytical and clinical validity of ctDNA MRD to identify ctDNA-positive patients who are at very high risk of recurrence, thus paving the way towards changing clinical practice to prevent recurrence and potentially de-escalate adjuvant treatment in ctDNA-negative patients.

## 6. Rational Combination Therapy: Challenges and Expectations

Despite the latest advances in surgery with preoperative and/or postoperative chemotherapeutic regimens such as PAXG and (or) mFOLFIRINOX, recurrence rates remain very high, ranging between 60–76% [8,63,98]. The main causes of the alarmingly high relapse are MRD after treatment and resistance to current chemotherapeutic regimens. Therefore, considering the successful results with neoadjuvant and adjuvant combination therapy in non-small cell lung cancer (NSLSC), melanoma, and triple negative breast cancer (TNBC) by adding molecular targeted therapy and ICIs [14,65,66,99], research efforts have been focused on the development of rational combination therapy to improve oncological outcomes in PDAC.

### 6.1. Molecularly Targeted Therapy

Following decades-long debate on the existence of CSCs [100], recent evidence on LGR5+ CSCs [82,101] and resistance to chemotherapy [102,103], particular interest has been concentrated on the development of targeted drugs and immunotherapeutic agents to successfully target CSCs. CSCs originate from non-malignant stem or progenitor cells, activating developmental signaling pathways. Thus, the inhibition of Wnt, Notch, Hedgehog (Hh), and Hippo has provided promising findings in preclinical studies and early-phase clinical trials. However, there is still a lack of evidence on the successful treatment of CSCs in phase III randomized trials [100,104,105,106].

Based on evidence on HRCs [85] in humanized mouse models and immune checkpoint blockade (ICB) treatment with anti-PD-1 and anti-CTLA-4 antibodies, the authors support neoadjuvant immunotherapy in patients with resectable CRC to eliminate MRD and prevent recurrence [85]. However, clinical trials in human cancer are required to confirm this hypothesis. Although evidence from clinical trials is still lacking, deeper insights could enable the discovery of novel methods to eliminate CSCs and prevent recurrence in the future.

Approximately 90% of PDACs harbor a KRAS mutation, and thus KRAS inhibitors could be an effective treatment option in patients with this mutation [24]. Although in the past decades KRAS has been considered undruggable [107], recent developments in bioengineering are providing the infrastructure of direct KRAS targeting, thus opening new therapeutic directions. Targeting KRAS p.Gly12Cys (G12C) in NSCLC has led to the two drugs sotorasib and adagrasib [108,109]. Early activity has been demonstrated in highly refractory malignancies such as PDAC with inhibitors targeting other variants [select REF from Nat Med paper]. G12D: Given that KRAS G12D accounts for approximately 50% of KRAS-mutated PDAC, a recent study explored the potential to inhibit the KRASG12D mutant protein with the non-covalent small molecule inhibitor MRTX1133 on early and advanced PDAC and its impact on the TME. This agent reverses early PDAC growth, increases intratumoral CD8+ effector T cells, decreases myeloid infiltration, and reprograms cancer-associated fibroblasts in mice. The authors conclude that a synergistic combination of MRTX1133 with ICB overall, which improved survival in treated mice, could successfully be translated into clinical trials [110].

### 6.2. Immunotherapy-Based Combinatorial Treatment

The discovery of PD-1 and CTLA-4 molecules several decades ago and the first approval of the CTLA-4 antibody ipilimumab fifteen years ago, and two years later of the anti-PD1 antibody nivolumab for metastatic melanoma, has transformed cancer immunotherapy research and clinical practice in solid tumors [111]. Recent advances in ICB treatment include not only clinical application in many metastatic solid tumors, but also in the perioperative setting of patients treated with curative intent. However, several challenges remain to be overcome. First, durable efficacy, with the exception of melanoma, has not yet been achieved in patients with metastasis at diagnosis. Second, the significantly improved EFS, DFS, RFS, cancer-specific survival, and OS are limited to immune-inflamed or hot tumors of patients with non-metastatic NSCLC, melanoma, TNBC, and mismatch repair-deficient (dMMR)/microsatellite instability-high (MSI-H) gastrointestinal cancers. Lastly, grade 3 and 4 adverse effects are approximately 30%, particularly in combination therapy.

In contrast to other solid tumors, ICIs have thus far been disappointing in the treatment of PDAC [11], demonstrating no clinical benefit in clinical studies [112,113,114]. Notably, in metastatic PDAC, for patients with tumours that are MSI-H/dMMR, are in the small proportion of patients with dMMR/MSI-H (1–3%), pembrolizumab produces durable benefit [115,116] based on KEYNOTE-158 and other studies that showed an overall response rate (ORR) ranging between 18–62%, leading to FDA approval of pembrolizumab in dMMR/MSI-H tumors including metastatic PDAC. Until multi-center multi-national powerful studies are published, there is still scepticism about whether ICIs can improve outcomes in other settings in PDAC. Clinical studies and trials in advanced and metastatic PDAC have resulted in ORRs of 0–3%. The spatiotemporal heterogeneity and dynamic evolution of the TME restrict intrinsic or therapy-induced antitumor immune responses in solid tumors. Molecular mechanisms underlying intrinsic resistance to ICIs in “immune-desert” or “cold” tumors, such as PDAC, include a lack of TILs within the tumor’s core and at its invasive margins due to physical barriers hindering their penetration of the stromal barrier [117,118]. Moreover, the presence of immunosuppressive cell populations such as tumor-associated macrophages (TAMs), myeloid-derived suppressor cells (MDSCs), and regulatory T cells (Tregs) and low immunogenicity [119] also contribute to the disappointing clinical results of ICIs in PDAC [11]. Therapeutic cancer vaccines that directly activate effector cells are emerging as potentially effective therapeutic approaches for immune-desert tumors [120].

In the emerging era of precision immuno-oncology [121,122,123], the personalized mRNA phase I clinical trial may transform research and clinical practice of PDAC patients undergoing surgical resection [18]. The German biotech company BioNTech, with expertise in fundamental research on mRNA technology and successful development and wide clinical use of mRNA vaccines in COVID-19 [124], and the Memorial Sloan Kettering (MSK) cancer center, with a focus on breakthrough research and clinical treatment on PDAC, have rapidly translated the personalized mRNA vaccines into a phase I clinical trial [18].

To overcome resistance to postoperative standard chemotherapy [38], the researchers were based on three pillars: (a) based on the mutation-derived T cell-specific neoantigens, they have manufactured the mRNA vaccine by RNA sequencing and whole-exome sequencing of the tumor, (b) Given that recent observations have shown that most PDACs in fact harbor more neoantigens [125,126,127] than previously predicted [128] as well as studies of long-term survivors of PDAC have revealed that neoantigens may stimulate T cells, strategies to deliver neoantigens may induce neoantigen-specific T cells and affect patient outcomes, and (c) Considering that evidence indicate that rational combination therapy in cancer and the synergy between autogene cevumeran with the an anti-PD-L1 antibody atezolizumab and adjuvant mFOLFIRINOX chemotherapy can overcome drug resistance, the authors realized this phase I randomized trial. The time period between production and administration of vaccines requires approximately 9 weeks, and therefore, combination therapy was initiated by atezolizumab, followed by autogene cevumeran and mFOLFIRINOX.

In the study, 16 patients were treated with the adjuvant combination therapy mentioned above. This adjuvant treatment has induced significant T cell activity. The T cell activity induced by autogene cevumeran was measured by ex vivo IFNγ ELISpot assay in the blood [18]. At a median follow-up of 18-months, 8 responder patients with expansion of T cells had longer RFS (overcoming 18 months) as compared to non–responders without T cell expansion (RFS 13.4 months) [*p* = 0.003]. The study holds great promise to reduce recurrence and improve oncological outcomes. The results of the ongoing phase II randomized trial on the efficacy and safety of adjuvant autogene cevumeran-based combinatorial treatment versus mFOLFIRINOX alone are awaited with notable interest.

### 6.3. Biomarkers to Guide Personalized Therapy

Despite the urgent need and intensive conventional research, the development of prognostic and predictive biomarkers to successfully prevent and treat solid tumors has not yet been fully achieved. At the beginning of this century, the completion of the first human genome sequencing was published [129,130], raising overenthusiasm for the personalized prevention and treatment of common diseases. At the same time, conventional clinicopathological characteristics and TNM classification [131], as well as single-gene sequencing for the identification of germline mutations, have been incorporated into clinical practice for the personalized prevention and treatment of solid tumors [13,132]. The advent of NGS in the market in 2006 has transformed biomedical and cancer research. Subsequently, the unprecedented potential for cancer genome sequencing in multiple clinical studies has revealed the extraordinary cancer genome evolution and heterogeneity, enabling a molecular taxonomy and the development of prognostic and predictive biomarkers to guide personalized treatment [133,134,135,136]. Three classes of static and dynamic biomarkers to guide personalization of combination therapy have recently emerged.

#### 6.3.1. Dynamic Longitudinal ctDNA MRD to Guide Individualized Treatment in High-Risk Patients

The unprecedented and unique potential of ctDNA MRD to predict recurrence and guide adjuvant treatment, as well as patient-surveillance, has attracted major clinical interest, translated into multiple phase II and III clinical trials. Rapid advances over the past decade in the development and validation of ctDNA-based MRD detection, referred to as ctDNA MRD, are based on tumor-informed and tumor-agnostic strategies.

Table 4 summarizes the results of observational studies and clinical trials in PDAC utilizing tumor-informed ctDNA approaches. The most reliable primary and secondary outcomes are sensitivity, specificity, DFS, RFS, and OS [137]. Observational studies are limited by extensive heterogeneity and small number of patients, while dynamic ctDNA analysis and sensitivity as the primary outcomes were done in only 3 studies (ranging between 48–57%) [138,139,140]. False-negative rates are also a strong limitation of existing studies. Most studies have made clear correlations regarding positive ctDNA status post-treatment and decreased survival [98,138,139,140,141,142,143,144,145]. To overcome these obstacles, one clinical trial has been completed [98], and another one has reported preliminary results (ACTRN12618000335291).

The DYNAMIC-Pancreas phase II/III RCT recently released preliminary results in 102 patients randomly assigned to de-escalation to shorter duration of mFOLFIRINOX) or standard of care adjuvant chemotherapy in post-surgery ctDNA-negative patients (ACTRN12618000335291). After a median follow-up of 36 months, 40% of patients had detectable ctDNA, and median RFS was 13 months, while in ctDNA-negative patients (33%), median RFS was 22 months. In the other trial, postoperative ctDNA positivity was strongly associated with recurrence (Table 4B).

Beyond tumor-informed ctDNA methods, tumor-agnostic approaches can be an alternative strategy. A comparative analysis of ctDNA MRD testing in CRC and breast cancer has shown greater sensitivity for tumor-informed approaches, while tumor-agnostic approaches offer broader applicability due to their reliance on plasma-only analysis and decreasing costs [95,96].

In PDA, tumor-agnostic strategies for ctDNA MRD testing have not yet been appropriately evaluated. In 33 patients with advanced PDA applying targeted DNA methylation sequencing, cfDNA fragmentomics, and machine learning. This strategy can estimate ctDNA levels, and multivariable Cox regression confirmed ctDNA levels as an independent predictor of PFS (HR1.9, *p* < 0.001) and OS (*p* < 0.001) [146].

#### 6.3.2. Comprehensive Genomic Profiling (CGP)-Based Assays to Guide Targeted Therapy

Advances in NGS-based whole-exome and whole-genome sequencing in clinical samples of solid tumors have led to the development and validation of multi-gene panels to identify actionable alterations for molecular targeted therapy. Recently, CGP with expanded gene panels, including more than 300 genes, have been approved by the US Food and Drug Administration (FDA), such as FoundationOneCDx and Illumina TruSight Oncology 500 (TSO500), to guide personalized therapy [147]. In PDAC, CGP has not yet been recommended for perioperative treatment of patients undergoing surgical resection; national guidelines recommend that an FDA-approved and/or validated NGS-based assay be used for patients with locally advanced/metastatic disease [38].

The unprecedented capacity of the TSO500 panel, including 523 genes, provides the unprecedented potential to identify clinically actionable and potentially actionable genomic alterations, including single-nucleotide variants (SNVs), short insertions and deletions (indels), copy number variants (CNVs), and gene fusions. More recently, the TSO500-based BULLET study has identified actionable mutations in not only high-frequency genes (KRAS and TP53) or middle frequency (CDKN2A), but also in 17 low-frequency (up to 20%) [148] in PDAC. This study, in combination with another retrospective analysis of 380 PDAC patients undergoing surgical resection with neoadjuvant and/or adjuvant chemotherapy using FDA-approved GCP assays [149], paved the way towards molecular targeted therapy beyond the metastatic setting into the perioperative setting.

#### 6.3.3. Deciphering the TME Towards Immunotherapy-Based Tailored Treatment

The crucial role of the TME in tumorigenesis, tumor growth, metastatic dissemination, and response to therapy is now well established in solid tumors [150,151]. The importance of the interplay of intracellular signaling network and the TME has been previously reported to understand TME complexity towards biomarker development [152]. The TME is comprised of cancer cells and the surrounding immune and stromal cells, as well as the ECM, with a plethora of dynamic interactions, underlying the complexity and aggressiveness of PDAC, and is reflected by the higher unresectable or metastatic disease at diagnosis. Moreover, additional complexity arises from the different TME components, such as innate antitumor immunity of cytotoxic CD8+ T cells, CD4+ helper T cells on one side, as well as the immunosuppressive part of the TME on the other side, including TAMs, MDSCs, Tregs, cancer-associated fibroblasts (CAFs), and ECM [22,153]. Additionally, the lack of TILs within the immune-desert PDAC TME and exhausted TME-resident T cells explains the intrinsic resistance to ICIs alone [154], indicating the urgent need for converting the immunologically ‘cold’ PDAC TME into a ‘hot’ TME [22].

The decoding of extreme complexity and aggressiveness, dynamically evolving PDAC TME, including spatiotemporal evolution of TME [136,155,156], heterogeneity, plasticity, and stem-like cancer cells on one side [157], as well as the immune landscape with the opposite functionality of CD8 and CD4 T cells as compared to TAMs, CAFs, MDSCs, and Treg, underlines resistance to systemic therapy [157]. The comprehensive concept of single-cell technologies-based development of a framework of biomarkers, such as holistic and dynamic TME analysis, as well as cell-free DNA (cfDNA) and ctDNA, has been previously reported [158,159]. More recently, the dramatic advances in single-cell multiomics, spatial transcriptomics and proteomics [21,160,161] and multidimensional big data analysis with AI are shaping a novel roadmap for realization of combination of tissue- and liquid biopsy-based biomarkers towards personalization of immunotherapy-based effective combinatorial treatment.

## 7. Future Directions

Over the next decade, the completion and publication of phase II and III RCTs, the gold standard of evidence-based medicine, coupled with the substantial advances in breakthrough research harnessing single-cell multiomics and spatial proteomics and transcriptomics [162,163], in combination with ctDNA MRD testing, is expected to improve oncological outcomes of PDAC [158]. Considering the concept of rational combination therapy [164,165,166] with successful integration in clinical practice for several other major solid tumors, such as NSCLC, breast cancer, and melanoma, in both perioperative and metastatic settings, current cutting-edge research focuses on the shift from standard chemotherapy to rational combination therapy. Rapid progress in the fields of immune-based combination therapy and biomarkers for tailored treatment holds great promise in the following issues.

First, it is time to shift from the more recent obsession with neoadjuvant and/or adjuvant updated chemotherapeutic regimens, supported by recent fundamental and translational discoveries, to reverse the disappointing estimates for the dramatic increase in incidence and mortality of PDAC for 2040 in high-income countries and worldwide [6]. Although the preoperative PAXG-based 3-year EFS was 33%, the pCR in the PAXG group was 3%, and in the mFOLFIRINOX group, 0%, suggesting the limitation of chemotherapy to eliminate MRD.

Second, the positive results of the phase I mRNA vaccine clinical trial have led to the large-scale (260 participants) phase II IMCODE003 RCT (NCT05968326), which evaluates the efficacy and safety of adjuvant autogene cevumeran plus atezolizumab and mFOLFIRINOX versus mFOLFIRINOX alone in participants with PDAC undergoing R0 or R1 resection. The primary outcome of the study is DFS, and secondary outcomes include OS at 3 and 5 years.

Third, the clinical validity of ctDNA MRD testing is being evaluated in three ongoing trials. Although the preliminary results in 102 out of 400 estimated patients are promising in the Dynamic-Pancreas ongoing trial, definite conclusions on the clinical validity and utility of ctDNA MRD guiding adjuvant chemotherapy have to be awaited (ACTRN12618000335291). Two other phase II interventional RCTs are ongoing (NCT05788744 and ChiCTR2000033479) as well as several observational and prospective studies. However, given that all patients with stage I-II-III PDAC undergoing surgical resection receive preoperative and/or postoperative chemotherapy [7,8], the clinical benefit of ctDNA MRD is limited to de-escalation of long-term chemotherapy or intensive imaging in ctDNA-negative or ctDNA-positive patients, respectively (Table 3B).

Therefore, considering the prime paradigm of rational combination therapy in other solid tumors [166], the ctDNA MRD testing in patients undergoing combination therapy could lead to improved EFS, RFS, and OS. Indeed, the ctDNA MRD testing as a prognostic biomarker to guide immunotherapy-based treatment is being evaluated in the ongoing adjuvant mRNA cancer vaccine trial, including patients with stage II-III CRC and ctDNA MRD-positive (NCT04486378). Moreover, an ongoing phase II RCT (AMPLIFY-7P) evaluates antitumor activity and clinical utility of adjuvant ELI-002 7P amphiphile peptide vaccine versus observation in patients with resected mutant KRAS PDAC who have completed standard-of-care treatment, considering also the ctDNA analysis as a biomarker (NCT05726864).

Fourth, beyond the KRAS-directed therapies focused on KRASG12D and RAS-GTP, CGP-based identification of actionable and potentially actionable alterations to guide targeted therapy, already recommended for the locally advanced/metastatic setting, the use of the most recently approved TSO500-based tailored targeted therapy holds promise to improve oncological outcomes in both metastatic and perioperative settings [19].

**Figure 2:**
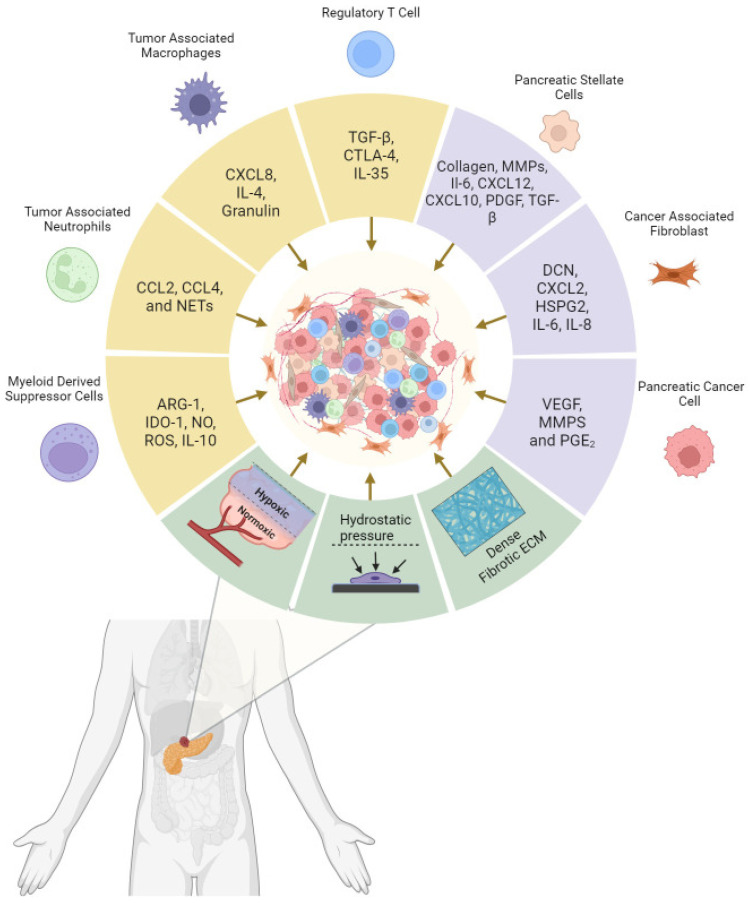
PDAC TME composition. Delineating the cancer cells and the surrounding immune (regulatory T cells, tumor-associated macrophages, tumor-associated neutrophils, and myeloid-derived suppressor cells) as well as cancer-associated fibroblasts and the extracellular matrix within the TME, the cytokines and signaling molecules they produce, and the pressures that occur, all resulting in a desmoplastic TME [167].

Fifth, the unique composition of the cold PDAC TME with a plethora of interactions between cancer cells and the suppressive part of TME, including CAFs, TAMs, Treg, MDSCs, and their interplay, is delineated in Figure 2 [167]. The efficacy of inactive, lack of, or exhausted CD8+ and CD4+ T cells in the immune-desert PDAC to destroy cancer cells through mRNA vaccine-based therapy [18,168] could further be improved by targeting the immunosuppressive CAFs, Treg, MDSCs, and their interactions [22,169]. The comprehensive understanding of the extraordinary complexity of PDAC TME to overcome resistance to systemic therapy remains enigmatic. However, spatial transcriptomics and, more recently, proteomics have revolutionized cancer research in a series of papers and an accompanying editorial in the Nature Methods journal [162,163,170]. The integration of single-cell multiomics (e.g., genomics, epigenomics, transcriptomics, and proteomics) [157] enables the unravelling of the TME heterogeneity of cancer cells and variability of immune landscape, suggesting the need for biomarker development to guide personalization of combination therapy [22,89,157,160,171,172].

Development of predictive biomarkers to guide mRNA cancer vaccine-based therapy represents a research priority. Although there are no details currently on the development of biomarkers to guide personalized autogene cevumeran-based treatment of PDAC patients in the phase II IMCODE003 RCT (NCT05968326), ctDNA MRD analysis could be a guide towards personalized therapy in high-risk ctDNA-positive patients and, at the same time, reduce the high cost of combination therapy in ctDNA-negative patients. Moreover, shifting from WES and RNA-sequencing in bulk samples [18,168] to single-cell genomics and transcriptomics in the manufacturing of mRNA cancer vaccine autogene cevumeran could enable the development of more effective personalized mRNA cancer vaccines.

Although single-cell approaches can identify cellular heterogeneity, they cannot provide the spatial organization of TME components [89,161]. Several innovative studies have improved our understanding of spatial tumour evolution and interactions between cancer cells with the local microenvironment in 3D space [156]. Innovation in spatial proteomics enables the characterization of proteomes while preserving spatial information, providing insights into tissue organization and cell–cell interactions [161]. A holistic approach harnessing spatial proteomics and transcriptomics, artificial intelligence, and systems biology can uncover a plethora of interacting TME components, including protein-protein interactions, molecular networks, and the cross-talk among immune and cancer cells, as well as CAFS and TAMs [161,171]. Given the interplay between various components within the PDAC TME, a multifaceted therapeutic approach targeting the network of TME interactions is essential to overcome the limitations of monotherapies [169]. Decoding the dynamics, heterogeneity, and interactions of TME components through spatial proteomics and transcriptomics in combination with genomics and metabolomics improves our comprehensive understanding of biological systems [21,160,161].

The emerging holistic 3D spatial localization, organization, and mapping of dynamic interactions between cancer cells and the surrounding immune and stromal cells, as well as the ECM, enable the comprehensive deciphering of dynamically evolving TME and mechanisms underlying therapeutic resistance [89,154,156,171,173,174,175,176]. This comprehensive understanding of 3D spatial architecture opens a new horizon in the development and validation of a framework of biomarkers guiding the multimodal therapy of the future. However, it is worth mentioning that the current technical limitations, high cost, and lack of standardization could be overcome in the near future through research funding, given the revolutionary nature of these high-precision technologies (single-cell multiomics, spatial transcriptomics and proteomics, AI, and systems biology).

Sixth, despite the great promises of mRNA vaccine-based therapy [18,168], recurrence due to intrinsic and acquired resistance remains challenging. The characterization of personalized mutational landscape-derived neoantigens activating specific CD8+ T cells subtypes is shaping the development of prognostic and predictive biomarkers towards patient-specific immunotherapy-based treatment. Potentially, the efficacy of adjuvant autogene cevumeran, atezolizumab, and mFOLFIRINOX [18,168] could be increased in future clinical trials evaluating neoadjuvant TSO500-based identification of actionable and potentially actionable alterations for molecularly targeted therapy added to current chemotherapeutic regimens followed by surgical resection and adjuvant mRNA vaccine-based treatment. Although the efficacy of the neoadjuvant approach has recently been demonstrated in phase III RCTs for immune-inflamed NSCLC and melanoma, this perioperative approach could also be effective in immune-desert PDAC, utilizing the latest advances in recently approved CGP assays for targeted therapy and immune-based treatment.

The holistic and dynamic TME analysis before neoadjuvant treatment through EUS-guided FNB to obtain sufficient material, as well as after surgical resection, can enable not only the deciphering of spatiotemporal evolution and dynamic changes of crucial TME components using high-precision profiling technologies mentioned above [172], but also could lead to the development of dynamic prognostic and predictive biomarkers guiding meaningful therapy. Moreover, the combination with longitudinal ctDNA MRD analysis could also contribute to the discovery of a novel framework of biomarkers guiding innovative multimodal therapy [177].

## 8. Conclusions

Advances in patient care of PDAC include screening and surveillance in patients with familial predispositions and germline mutations. Moreover, EUS-guided FNB, standardization of surgical resection, and the recent trend in perioperative treatment demand multidisciplinary consultation of experts at high-volume centers, enabling increased resectability and complete surgical resection. Despite this progress, the majority of patients are diagnosed with unresectable or metastatic disease (~80%), while recurrence rates remain alarmingly high even with recent perioperative chemotherapy. It is not surprising that the estimates of incidence and mortality for 2040 by the WHO are disappointing, with PDAC having the worst CFR.

In contrast to this prediction, the rapidly evolving fundamental and translational research in mRNA cancer vaccines and ctDNAMRD assays are currently being evaluated in ongoing RCTs, holding promises to transform clinical practice. Furthermore, comprehensive spatiotemporal analysis of TME before and after preoperative treatment, as well as longitudinal ctDNA MRD, thus holds great promise to improve EFS, RFS, cancer-specific survival, and OS rates in the foreseeable future. Integrating single-cell multiomics, spatial proteomics and transcriptomics, artificial intelligence, and systems biology algorithms in tertiary institutions with expertise in innovative research opens new predictive and therapeutic avenues. These high-precision technologies, revolutionizing cancer research, enable the holistic exploration of 3D spatial architecture of the crucial and interacting TME components. Harnessing biospecimens analysis with these cutting-edge technologies before preoperative treatment and after surgical resection will uncover the dynamic changes of TME, thus enabling the development and validation of TME-based prognostic and predictive biomarkers at baseline and following treatment. Considering the extraordinary complexity of TME ecosystem and the high intrinsic and acquired resistance, even after adjuvant mRNA vaccine-based therapy and the advantages of preoperative therapy, neoadjuvant modern chemotherapy and TSO500-based molecular targeted therapy in the proportion of patients harboring targetable mutations, justifies small phase I trials evaluating the efficacy and safety of this perioperative combination therapy. A comprehensive and dynamic TME-, TSO500- and ctDNA MRD-based framework of prognostic and predictive biomarkers is opening the new horizon of precision immuno-oncology and oncology. Translating this framework of three biomarkers, enabling an optimal patient-specific rational multimodal treatment that includes perioperative precision immunotherapy, molecular targeted drugs, and modern chemotherapy, within next-generation clinical trials, paves the way towards the elimination of MRD and disease relapse prevention. Beyond multidisciplinary consultation of experts at high-volume centers, the management of the disease, international cooperation in the shaping of rational roadmap, including researchers with expertise in high-precision technologies exploring spatial architecture and temporal evolution of the PDAC TME ecosystem, is also needed towards a patient-specific meaningful therapy to improve the outcomes of patients with PDAC.

Decisions about diagnosis, resectability, and management should involve multidisciplinary consultation at a high-volume center with use of appropriate imaging studies.

## Figures and Tables

**Table 1:** Incidence and mortality estimations in 2022 and projections for 2040 for pancreatic cancer according to the Global Cancer Observatory in absolute numbers and percent change worldwide and in different geological areas.

Region	Year	Incidence/Mortality	Number of Cases/Deaths (+ % Change from 2022 to 2040)
Europe	2022	Incidence	146.000
		Mortality	139.000
	2040	Incidence	181.000 (+24%)
		Mortality	174.000 (+25.2%)
USA	2022	Incidence	60.100
		Mortality	49.500
	2040	Incidence	82.500 (+37.2%)
		Mortality	69.900 (+42.2%)
China	2022	Incidence	119.000
		Mortality	106.000
	2040	Incidence	189.000 (+58.8%)
		Mortality	174.000 (+64.2%)
Japan	2022	Incidence	47.600
		Mortality	43.300
	2040	Incidence	53.000 (+11.3%)
		Mortality	49.200 (+13.6%)
Global	2022	Incidence	511.000
		Mortality	467.000
	2040	Incidence	820.000 (+60.5%)
		Mortality	761.000 (+63%)

**Table 2:** Case fatality rate (CFR) range globally and in very high Human Development Index (HDI) countries in 2022 and 2040, according to WHO, among the five leading cancer types.

Cancer Type	2022 CFR (Cases/Deaths)		2040 CFR (Cases/Deaths)	
	Global	Very High HDI Countries ^1^	Global	Very High HDI Countries ^1^
1. Pancreas	1.09	1.10 [From 1.03 in Germany to 1.21 in USA]	1.08	1.08 [From 1.00 in The Netherlands and Canada to 1.18 in USA]
2. Liver	1.14	1.22 [From 1.02 in Turkey to 1.57 Japan]	1.13	1.18 [From 1.00 in Turkey to 1.43 in Japan]
3. Lung	1.36	1.40 [From 1.06 in Turkey and Mexico to 1.77 in USA]	1.33	1.36 [From 1.02 in Turkey to 1.66 in USA]
4. Colorectal	2.13	2.31 [From 1.85 in Turkey to 3.00 in Australia]	2.02	2.13 [From 1.66 in Canada to 3.14 in Denmark]
5. Breast	3.45	4.45 [From 3.42 in Turkey to 6.46 in Australia]	3.24	4.01 [From 3.04 in Turkey to 6.62 in Republic of Korea]

^1^ Including 18 of the countries with very high HDI: Iceland, Norway, Switzerland, Denmark, Germany, Sweden, Australia, The Netherlands, UK, Canada, USA, Japan, Republic of Korea, Spain, France, Italy, Russia, Turkey, and Mexico. Abbreviations: CFR, case fatality rate; HDI, human development index.

**Table 3:** Selected completed (**A**) and ongoing (**B**) clinical trials for neoadjuvant therapy of non-metastatic stage I-III PDAC.

Study	Patients	Disease Stage	Regimen	Endpoint
**A. COMPLETED**				
**CASSANDRA [8]** 2020–2024 Phase III Randomized	260	R/BR	NA PAXG vs. NA mFOLFIRINOX	Median EFS: 16 months vs. 10.2 months 3-year EFS: 31% vs. 15% pCR: 3% vs. 0%
**PREOPANC1 [62]** 2013–2017 Phase III Randomized	246	R/BR	NA gemcitabine-based chemoradiotherapy vs. upfront surgery	5-year OS: 20.5% vs. 6.5%
**ESPAC5 [48]** Phase II Randomized 2014–2018	478 patients	BR	Four groups: 1. Immediate surgery, 2. NA gemcitabine and capecitabine, 3. NA FOLFIRINOX and 4. NA capecitabine-based chemoradiation	1-year OS: 39% for arm 1, 78% for arm 2, 84% for arm 4 1-year DFS: 33% for immediate surgery and 59% for the combined neoadjuvant therapies
**Alliance A021501 [49]** Phase II Randomized 2016–2020	126 patients	BR	NA mFOLFIRINOX plus radiation (SBRT or hypofractionated) vs. NA mFOLFIRINOX only	18-month OS rate 66.7% vs. 47.3%
**PANACHE01-PRODIGE 48 [50]** Phase II Randomized 2017–2021	153	R	NA mFOLFIRINOX vs. NA FOLFOX vs. upfront surgery	1-year OS: Upf. surgery = 80.8%, mFOLFIRINOX = 84.1% & FOLFOX = 71.8% 1-y EFS: Upf. surgery = 38.7%, mFOLFIRINOX = 51.4% & FOLFOX = 43.1%
**NORPACT1 [51]** Phase II Randomized 2016–2022	140	R	NA mFOLFIRINOX followed by surgery and adjuvant mFOLFIRINOX vs. upfront surgery and adjuvant mFOLFIRINOX	18-month OS rate 57% vs. 70%
**NUPAT-01 [52]** Phase II Randomized	51	BR	NA FOLFIRINOX vs. NA GEM/nab-PTX	3-year OS: 54.7% median OS: 39.4 mo Significant difference in OS was **not** observed between the FOLFIRINOX and GEM/nab-PTX groups.
**Prep-02/JSAP-05 [53]** Phase II/III Randomized 2013–2019 Tohoku University Japan	362	R	NA GEM + S-1 vs. Upfront Surgery (4 courses adjuvant S-1 for both arms)	Median OS: 37 months vs. 26.6 months Median RFS: 14.3 months vs. 11.3 months
**SWOG S1505 [54]** Phase II Randomized 2016–2020	147	R	NA mFOLFIRINOX vs. NA GEM/nab-PTX	2-year OS: 47% with mFOLFIRINOX and 48% with GEM/nab-PTX (Not significant)
**Alliance A021101 [55]** Phase II Non-randomized 2013–2018	22	BR	NA mFOLFIRINOX + capecitabine-based chemoradiation followed by surgery and adjuvant gemcitabine	Median OS: 21.7 months
Nagakawa et al. 2017 [56] Phase II Non-Randomized	27	BR	NA gemcitabine-based chemoradiation	Median OS 22.4 mo 1-year OS 81.3% 2 year OS 33.9%
**SMART [57]** Phase II Single arm	136	BR/LA	Induction FOLFIRINOX or GEM/nab-PTX followed by SBRT 50 Gy/5 (if no disease progression)	1-year PFS: 46% 1-year LC: 78% 1-year OS: 56%
Jeon et al. 2022 [58] 2017–2019 Retrospective study	64	BR/LA/m	NA FOLFIRINOX	pCR = 12.5% npCR = 12.5%
Tamburrino et al. 2024 [59] Retrospective study	403	R/BR	NA chemotherapy followed by surgery and standard adjuvant chemotherapy	pCR = 3.8% 3-y disease-specific survival = 87% in pCR patients vs. 43% in npCR (*p* = 0.014) Recurrence = 40% (*n* = 6/15) in pCR group vs. 69.8% (*n* = 271/388) in npCR. 1-y DFS = 80% (pCR group) vs. 60% (npCR group) 3-y DFS = 48% (pCR group) 24% (npCR group)
**NEONAX [60]** Phase II Randomized 2015–2022	127	R	Perioperative GEM/nab-PTX vs. adjuvant only GEM/nab-PTX	18-month DFS: 33.3% vs. 41.4% Median OS: 25.5 vs. 16.7 months
Jang et al. [61] Phase II/III Randomized 2018	58	BR/LA	NA gemcitabine-based Chemoradiation vs. upfront Surgery (adjuvant GEM for both)	2-year survival rate: 40.7% (21 months) vs. 26.1% (12 months)
**B. ONGOING**				
**PREOPANC-3** Ongoing Phase III Randomized (2021–2029) NCT04927780	378 (Estimated)	R	8 cycles NA mFOLFIRINOX followed by surgery and 4 cycles adjuvant mFOLFIRINOX vs. Surgery and adjuvant 12 cycles mFOLFIRINOX	Primary: OS (Up to 5 years after randomization) Secondary: PFS, DMFS, Locoregional PFS, pCR, R0 rate
**Alliance A021806** Ongoing Phase III Randomized (2020–2030) NCT04340141	352 (estimated)	R	8 cycles NA mFOLFIRINOX followed by surgery and 4 cycles adjuvant mFOLFIRINOX vs. Surgery and adjuvant 12 cycles mFOLFIRINOX	Primary: OS (up to 6 years) Secondary: 6-year DFS, Time to locoregional recurrence, Time to distant metastases, pCR, R0 rate
**NeoFOL-R** Ongoing Phase III Randomized (2023–2027) NCT05529940	609 (estimated)	R	6 cycles of NA mFOLFIRINOX followed by surgery and 6 cycles adjuvant mFOLFIRINOX vs. Surgery and adjuvant 12 cycles mFOLFIRINOX	Primary: 2-year survival rate Secondary: 5-year OS, 3 years DFS, 5-year OS, 3 years DFS, Local recurrence rate
**PANDAS-PRODIGE 44** Ongoing Phase II Randomized (2016–2027) NCT02676349	130	BR	NA mFOLFIRINOX + capecitabine-based chemoradiotherapy vs. NA mFOLFIRINOX (surgery and adjuvant chemotherapy in both)	R0 resection margin rate (at 7.5 months): – -
**STEREOPAC** Ongoing Phase II Randomized (2023–2030) NCT05083247	256 (estimated)	BR	4 cycles of mFOLFIRINOX + 4 additional cycles of chemo followed by surgery vs. 4 cycles of mFOLFIRINOX + 5th and 6th cycles of chemo then iHD-SBRT followed by a 7th (and optional 8th cycle) followed by surgery	R0 resection at 12 months and 100 weeks DFS - -
Medical College of Wisconsin Phase II Randomized (2018–2024) NCT03704662	102 (estimated)	R/BR/LA	NA SBRT vs. Conventional concurrent chemotherapy and radiation therapy	The number of subjects who present with node-positive disease following surgical resection (8 weeks post-radiation)
**AGITG Masterplan** Phase II Randomized 2019 ACTRN12619000409178	120	R/BR/LA	NA mFOLFIRINOX vs. Neoadjuvant mFOLFIRINOX followed by SBRT and mFOLFIRINOX (Surgery and adjuvant mFOLFIRINOX or GEM/CAP in both)	LC 1 year
**PREOPANC-5** Phase I/II 2024–2028 NCT06384560	66	BR	NA Triple Treatment (FOLFIRINOX, SABR and pembrolizumab)	18-month PFS
**NeoPancOne** Phase II 2020–2025 NCT04472910	84	R	NA mFOLFIRINOX up to 6 cycles followed by surgery and adjuvant chemotherapy for up tp 6 cycles	2–4 years DFS assessment according to baseline GATA6 expression level

Abbreviations: R, resectable; BR, borderline resectable; LA, locally advanced; NA, neoadjuvant; EFS, event-free survival; OS, overall survival; pCR, pathological complete response; npCR, non-pCR; DFS, disease-free survival; RFS, recurrence-free survival; PFS, progression-free survival; LC, local control; DMFS, distant metastasis-free survival; GEM/nab-PTX, gemcitabine/nab-paclitaxel; SBRT, Stereotactic Body Radiation Therapy; iHD-SBRT, Isotoxic High-Dose SBRT; SABR, Stereotactic Ablative Radiotherapy; GEM/CAP, gemcitabine/capecitabine.

**Table 4:** Observational studies and clinical trials of ctDNA MRD in pancreatic cancer with published results (**A**) and ongoing (**B**).

Study Details	Cancer Type and Stage	Intervention and Description	ctDNA Information	Main Outcome	Published Results
**A. With published results**					
1. Dickey EM et al. 2025 [138] Observational	Stage pT1-4N0-2M0 PDAC 32 patients	Completion of all curative-intent therapy, 24 patients underwent neoadjuvant chemotherapy (mostly FOLFIRINOX)	Surveillance (Pre-operative ctDNA was excluded from analysis) ctDNA testing was performed via SignateraTM platform (tumor-informed) at various time points following completion of all standard therapies	RFS	ctDNA positivity rate: 28.1% median follow-up: 17.7 months mRFS significantly lower in patients with positive ctDNA (3.6 vs. 29.0 months, *p* < 0.001) Sensitivity: 47.4% Specificity: 100% PPV: 100% NPV: 56.5%
2. Botta GP et al. 2024 [141] Observational retrospective study (2019–2023)	Stage I-III resectable & borderline-resectable PDAC 298 patients	SoC 47% (140 patients) receiving neoadjuvant treatment	Perioperative blood samples and during surveillance tumor-informed, 16-plex mPCR NGST assay (SignateraTM)	DFS	Median follow-up: 13 months In the MRD window (within 2–12 weeks of surgery, prior to therapy): Positive ctDNA detection rate = 29% mDFS = 6.37 months for ctDNA+ vs. 33.31 months for ctDNA- In the surveillance window (>12 weeks post-surgery, if no ACT was given, or starting 4 weeks post-ACT): - ctDNA detection rate = 29.6% - mDFS = 11.4 months for ctDNA+ vs. NR for ctDNA-
3. Hata T et al. 2023 [142] Observational	Resectable PDAC 66 patients	SoC	Preoperative and paired postoperative blood samples Cell-free DNA was extracted from the plasma, and KRAS mutations, as a benchmark of ctDNA, were examined using droplet digital PCR (tumor-informed)	DFS and OS in ctDNA+ vs. ctDNA-	Worst DFS and OS in ctDNA+, Independent risk factor for recurrence
4. Jiang J et al. 2020 [122] Observational	Stage I-II and IV PDAC 27 patients	SoC	preoperation, postoperation (7-days after surgery), and each follow-up visits (1 or 3 months after operation) Tumor informed ctDNA assay (matched tumor tissue to detect mutations)	DFS	Patients with ctDNA+ status postoperatively had a markedly reduced DFS compared to those with ctDNA-negative status (*p* = 0.019) Pre-operative: 66.7% ctDNA+ Postoperative ctDNA was positive in 9 patients, and 8/9 patients ultimately recurred.
5. Lee B et al. 2019 [139] Observational prospective	Localized PDAC 38 patients with KRAS mutation	SoC	Pre- and post-operative samples for ctDNA analysis were collected. PCR-based-SafeSeqS assays were used to identify mutations in KRAS (tumor-informed)	CA19–9 levels, RFS and OS for ctDNA status	ctDNA was detected in 62% (23/37) pre-operative and 37% (13/35) post-operative cases. 13/13 (100%) with ctDNA+ had recurrence
6. Kitahata Y et al. 2021 [140] Observational (2017–2020)	Resectable and borderline-resectable PDAC. 97 patients	SoC	pre- and postoperative plasma samples (Tumor-informed)	Association of ctDNA status and RFS and OS	pre- and postoperative ctDNA = in 9 patients, and neither was detected in 55 patients. Whereas 15 patients harbored only preoperative ctDNA, 18 patients had only post-op ctDNA. Pre-op ctDNA+ = poorerOS (*p* = 0.008) and post-op ctDNA+ = not associated with either RFS or OS
7. Lee Y et al. 2022 [145] Observational	Resectable Stage IA-III PDAC 53 evaluable	SoC	parallel analysis of pre- and postoperative ctDNA and matched tumor tissues using CAPP-Seq targeting 77 genes.	ctDNA levels (he/mL) RFS	Preoperative ctDNA = 37.7% of patients (20) Out of these, 12 (22.6% of total patients) still had detectable ctDNA after curative resection Tendency toward shorter RFS at 1 year was observed for patients with postoperative ctDNA > 1 hGE/mL
8. Cecchini M et al. 2024 [98] Phase II non-randomized (2014–2021)	Resectable PDAC 46 patients	Perioperative mFOLFIRINOX	tumor-informed ctDNA assay (SignateraTM) 16 somatic single-nucleotide variants	PFS	12-month PFS: 67%, mPFS = 16.6 months, mOS = 37.2 months. Baseline ctDNA levels were detected in 16 of 22 patients (73%) and in 3 of 17 (18%) after 6 cycles of mFOLFIRINOX. ctDNA+ had worse PFS and OS (significant)
**B. Ongoing**					
1. ARTEMIS-PC Observational (2022–2026) NCT06043921	Stage I-IV Resectable (50 patients) and unresectable (100 patients) PDAC 150 estimated patients	SoC	Resectable cohort: blood samples before surgery and at 1, 3, 6, 9, 12, 18, and 24 months after surgery Unresectable: blood samples before treatment and at 4, 8, 12, 16, 24, 32, 40, and 48 weeks on treatment	3-years: Unresectable cohort: Rate of concordance of KRAS mutations between tumor tissue and blood samples & pretreatment ctDNA detection rate for each disease stage; Resectable cohort: Success rate of WES assays and selections of personalized genes using biopsy tissue specimens & rate of ctDNA positivity for each cancer stage before neoadjuvant chemotherapy and 4 weeks after surgery.	21 pts with resectable PDAC and 64 patients with unresectable have been enrolled as of September 2023
2. Ruijin Hospital MRD study Observational prospective (2023) NCT05479708	Stage I-III PDAC with KRAS mutations 150 estimated patients	SoC	Post-surgery ctDNA detection	3-year OS	-
3. GUIDE.MRD Observational (2024) NCT06102889	Resectable PDAC 200 estimated patients	SoC	blood samples for multiple analysis in genomics, proteomics, and metabolomics	1 to 24 months post-surgery recurrence	-
4. ORACLE Observational (2021) NCT05059444	Many cancer types: Cohort 7: resected or resectable PDAC 1050 estimated patients	SoC	Landmark (+blood samples at standard of care and follow-up)	3-year Distant Recurrence Free Interval	-
5. Fudan University MRD study Observational (2023) NCT06151691	Stage I-II PDAC 51 estimated patients	SoC	Landmark (+blood samples after surgery and after adjuvant therapy) methylation, fragment group, and CNV multi-omics testing	18-month DFS	-
6. ADAPT-MRD Interventional randomized (2025) NCT06966440	Stage I-III PDAC with R0 resection 856 estimated patients	Adjuvant chemotherapy Experimental: Arm A: MRD-guided adjuvant treatment Placebo Comparator: Arm B: non-MRD-driven, standard of care adjuvant treatment	MRD testing will be performed before the start of the first treatment cycle, and at weeks 8 and 11 of each subsequent cycle Tumor-informed NGS	3-year DFS	-
7. Ruijin Hospital MRD study Observational (2025) NCT07080021	Borderline resectable and locally advanced PDAC patients undergoing standard NAC 119 estimated patients	SoC	Dynamic ctDNA MRD monitoring by serial blood draws at predefined timepoints	1-year correlation between ctDNA-MRD status at serial monitoring points during neoadjuvant therapy and therapeutic efficacy (R0 resection) and survival	-
8. DYNAMIC-Pancreas Phase II/III randomized (2019) ACTRN12618000335291	Stage I-III PDAC 438 patients	Post-curative resection randomization to ctDNA-directed adjuvant treatment (ctDNA positive recommended escalation with gemcitabine doublet therapy, ctDNA negative de-esclatation to shorter duration of mFOLFIRINOX) or SoC adjuvant chemotherapy	Landmark Tumor-informed ctDNA assay	2-year RFS	102 patients enrolled so far, R0 rate: 77% post-resection: 40% ctDNA+ & 33% ctDNA- (4% no result) In ctDNA- patients: 44% de-escalated to planned 3 months of AC 36 months follow-up, mRFS = 13 months in ctDNA+ vs. 22 months in ctDNA- (*p* = 0.003)
9. CIRCPAC Phase II randomized (2023) NCT05788744	Stage I-II PDAC 700 estimated patients	ctDNA guided surveillance; ctDNA positive patients will be randomized between active surveillance and SoC surveillance. In the active surveillance arm ctDNA positivity will trigger intensified imaging	Landmark & surveillance	3-year: ctDNA detection, levels, ctDNA DFS, ctDNA OS	-
10. AMPLIFY 7P Phase I/II randomized (2023) NCT05726864	Resected PDAC, 158 estimated patients	SoC followed by adjuvant ELI-002 7P vaccine (a lipid-conjugated oligonucleotide plus a mixture of lipid-conjugated peptide-based antigens)	Baseline (after completion of SoC and before vaccine) & After vaccine	1-year DFS, 6-months ctDNA reduction or clearance, 20-week OS	-
11. Fudan University MRD study Phase II non-randomized (2021) ChiCTR2000033479	Stage I-III PDAC 60 estimated patients	Gemcitabine, paclitaxel post-curative resection ctDNA positive: treatment with gemcitabine and paclitaxel, ctDNA negative: treatment with gemcitabine	Landmark	DFS	-

Abbreviations: PDAC, pancreatic ductal adenocarcinoma; ctDNA; circulating tumor DNA; RFS, recurrence-free survival; PPV, positive predictive value; NPV, negative predictive value; SoC, standard of care; PCR, polymer chain reactions; mPCR, multiplex PCR; NGS, next generation sequencing; DFS, disease-free survival; MRD, minimal residual disease; ACT, adjuvant chemotherapy; mDFS, median disease-free survival; OS, overall survival; PFS, progression-free survival; CAPP-seq, cancer personalized profiling by deep Sequencing; WES, whole exome sequencing; CNV, copy number variation; NAC, neoadjuvant chemotherapy.

## Data Availability

No new data were created or analyzed in this study.

## Abbreviations

The following abbreviations are used in this manuscript:

PDAC	pancreatic ductal adenocarcinoma
CFR	case fatality rate
PAXG	cisplatin, nab-paclitaxel, capecitabine, and gemcitabine
mFOLFIRINOX	5-fluorouracil with leucovorin, irinotecan, and oxaliplatin
ICIs	immune checkpoint inhibitors
NGS	next-generation sequencing
CGP	comprehensive genomic profiling
ctDNA	circulating tumor DNA
MRD	minimal residual disease
RFS	recurrence-free survival
OS	overall survival
SC	single-cell
MO	multiomics
SPT	spatial proteomics and transcriptomics
AI	artificial intelligence
TME	tumor microenvironment
WHO	World Health Organization
EFS	event-free survival
CSCs	cancer stem cells
ECM	extracellular matrix
NCI	National Cancer Institute
SEER	Surveillance, Epidemiology, and End Results
HDI	Human Development Index
PanIN	pancreatic intraepithelial neoplasia
IPMN	intraductal papillary mucinous neoplasm
WGS	Whole-genome sequencing
MRI	magnetic resonance imaging
MRCP	magnetic resonance cholangiopancreatography
EUS	endoscopic ultrasound
MPD	pancreatic duct diameter
PD	pancreatoduodenectomy
DPPHR	duodenum-preserving pancreatic head resection
CT	computed tomography
FNB	fine needle biopsy
FNA	fine needle aspiration
BR	borderline resectable
LA	locally advanced
RCT	randomized clinical trial
pCR	complete pathologic response
MPR	major pathologic response
pPR	partial pathologic response
pNR	pathologic non-response
R	resectable
NA	neoadjuvant
DMFS	distant metastasis-free survival
PFS	Progression-free survival
LC	local control
GEM/nab-PTX	gemcitabine/nab-paclitaxel
SBRT	stereotactic Body Radiation Therapy
iHD-SBRT	isotoxic High-Dose SBRT
SABR	stereotactic Ablative Radiotherapy
GEM/CAP	gemcitabine/capecitabine
ESMO	European Society For Medical Oncology
PET	positron emission tomography
ITH	intratumor heterogeneity
CRC	colorectal cancer
HRC	high-relapse cells
VAFs	variant allele frequencies
CTCs	circulating tumor cells
NSLSC	non-small cell lung cancer
TNBC	triple negative breast cancer
Hh	Hedgehog
ICB	immune checkpoint blockade
dMMR	mismatch repair deficient
MSI-H	microsatellite instability-high
ORR	overall response rate
TAMs	tumor-associated macrophages
MDSCs	myeloid-derived suppressor cells
Tregs	regulatory T cells
MSK	Memorian Sloan Kettering
PPV	positive predictive value
NPV	negative predictive value
SoC	standard of care
PCR	polymer chain reactions
mPCR	multiplex PCR
ACT	adjuvant chemotherapy
mDFS	median DFS
CAPP-seq	cancer personalized profiling by deep Sequencing
WES	whole exome sequencing
NAC	neoadjuvant chemotherapy
FDA	US Food and Drug Administration
TSO500	Illumina TruSight Oncology 500
SNVs	single nucleotide variants
indels	short insertions and deletions
CNVs	copy Number variants
CAFs	cancer-associated fibroblasts
cfDNA	cell-free DNA
TAN	tumor-associated neutrophil
DC	dendritic cells
NK cells	natural killer cells

## References

[1] Li D., Xie K., Wolff R., Abbruzzese J.L. (2004). Pancreatic Cancer. Lancet.

[2] Huang L., Jansen L., Balavarca Y., Molina-Montes E., Babaei M., van der Geest L., Lemmens V., Van Eycken L., De Schutter H., Johannesen T.B. (2019). Resection of Pancreatic Cancer in Europe and USA: An International Large-Scale Study Highlighting Large Variations. Gut.

[3] Dekker E.N., van Dam J.L., Janssen Q.P., Besselink M.G., DeSilva A., Doppenberg D., van Eijck C.H.J., Nasar N., O’Reilly E.M., Paniccia A. (2024). Improved Clinical Staging System for Localized Pancreatic Cancer Using the ABC Factors: A TAPS Consortium Study. J. Clin. Oncol..

[4] Siegel R.L., Kratzer T.B., Wagle N.S., Sung H., Jemal A. (2026). Cancer Statistics, 2026. CA Cancer J. Clin..

[5] Mackay T.M., Latenstein A.E.J., Augustinus S., van der Geest L.G., Bogte A., Bonsing B.A., Cirkel G.A., Hol L., Busch O.R., den Dulk M. (2024). Implementation of Best Practices in Pancreatic Cancer Care in the Netherlands: A Stepped-Wedge Randomized Clinical Trial. JAMA Surg..

[6] Ferlay J., Ervik M., Lam F., Colombet M., Mery L., Pineros M., Znaor A., Soerjomataram I., Bray F. Global Cancer Observatory Cancer Today. International Agency for Research on Cancer, Lyon. References—Scientific Research Publishing. https://www.scirp.org/reference/referencespapers?referenceid=2909542.

[7] Conroy T., Castan F., Lopez A., Turpin A., Ben Abdelghani M., Wei A.C., Mitry E., Biagi J.J., Evesque L., Artru P. (2022). Five-Year Outcomes of FOLFIRINOX vs Gemcitabine as Adjuvant Therapy for Pancreatic Cancer: A Randomized Clinical Trial. JAMA Oncol..

[8] Reni M., Macchini M., Orsi G., Procaccio L., Malleo G., Carconi C., Rapposelli I.G., Bencardino K., Scartozzi M., Balzano G. (2026). Preoperative mFOLFIRINOX versus PAXG for Stage I-III Resectable and Borderline Resectable Pancreatic Ductal Adenocarcinoma (PACT-21 CASSANDRA): Results of the First Randomisation Analysis of a Randomised, Open-Label, 2 × 2 Factorial Phase 3 Trial. Lancet.

[9] Finan J.M., Guo Y., Goodyear S.M., Brody J.R. (2024). Challenges and Opportunities in Targeting the Complex Pancreatic Tumor Microenvironment. JCO Oncol. Adv..

[10] Stoop T.F., Javed A.A., Oba A., Koerkamp B.G., Seufferlein T., Wilmink J.W., Besselink M.G. (2025). Pancreatic Cancer. Lancet.

[11] Ullman N.A., Burchard P.R., Dunne R.F., Linehan D.C. (2022). Immunologic Strategies in Pancreatic Cancer: Making Cold Tumors Hot. J. Clin. Oncol..

[12] Roukos D.H. (2000). Current Status and Future Perspectives in Gastric Cancer Management. Cancer Treat. Rev..

[13] Roukos D.H., Briasoulis E. (2007). Individualized Preventive and Therapeutic Management of Hereditary Breast Ovarian Cancer Syndrome. Nat. Clin. Pract. Oncol..

[14] Zhou F., Guo H., Xia Y., Le X., Tan D.S.W., Ramalingam S.S., Zhou C. (2025). The Changing Treatment Landscape of EGFR-Mutant Non-Small-Cell Lung Cancer. Nat. Rev. Clin. Oncol..

[15] Hortobagyi G.N. (2005). Trastuzumab in the Treatment of Breast Cancer. N. Engl. J. Med..

[16] Roukos D.H., Murray S., Briasoulis E. (2007). Molecular Genetic Tools Shape a Roadmap towards a More Accurate Prognostic Prediction and Personalized Management of Cancer. Cancer Biol. Ther..

[17] Roukos D.H. (2010). Targeting Gastric Cancer with Trastuzumab: New Clinical Practice and Innovative Developments to Overcome Resistance. Ann. Surg. Oncol..

[18] Rojas L.A., Sethna Z., Soares K.C., Olcese C., Pang N., Patterson E., Lihm J., Ceglia N., Guasp P., Chu A. (2023). Personalized RNA Neoantigen Vaccines Stimulate T Cells in Pancreatic Cancer. Nature.

[19] Saito Y., Horie S., Kogure Y., Mizuno K., Ito Y., Mizukami Y., Kim H., Tamura Z., Koya J., Funakoshi T. (2026). Real-World Clinical Utility of Comprehensive Genomic Profiling in Advanced Solid Tumors. Nat. Med..

[20] Montagut C., Vidal J. (2019). ctDNA to Detect Minimal Residual Disease in Pancreatic Cancer: Moving into Clinical Trials. Ann. Oncol..

[21] De Visser K.E., Joyce J.A. (2023). The Evolving Tumor Microenvironment: From Cancer Initiation to Metastatic Outgrowth. Cancer Cell.

[22] Kung H.-C., Zheng K.W., Zimmerman J.W., Zheng L. (2025). The Tumour Microenvironment in Pancreatic Cancer—New Clinical Challenges, but More Opportunities. Nat. Rev. Clin. Oncol..

[23] Hughes T., Harper A., Gupta S., Frazier A.L., van der Graaf W.T.A., Moreno F., Joseph A., Fidler-Benaoudia M.M. (2024). The Current and Future Global Burden of Cancer among Adolescents and Young Adults: A Population-Based Study. Lancet Oncol..

[24] Astiazaran-Symonds E., Goldstein A.M. (2021). A Systematic Review of the Prevalence of Germline Pathogenic Variants in Patients with Pancreatic Cancer. J. Gastroenterol..

[25] De Bruijn I., Kundra R., Mastrogiacomo B., Tran T.N., Sikina L., Mazor T., Li X., Ochoa A., Zhao G., Lai B. (2023). Analysis and Visualization of Longitudinal Genomic and Clinical Data from the AACR Project GENIE Biopharma Collaborative in cBioPortal. Cancer Res..

[26] Muraki T., Jang K.-T., Reid M.D., Pehlivanoglu B., Memis B., Basturk O., Mittal P., Kooby D., Maithel S.K., Sarmiento J.M. (2022). Pancreatic Ductal Adenocarcinomas Associated with Intraductal Papillary Mucinous Neoplasms (IPMNs) versus Pseudo-IPMNs: Relative Frequency, Clinicopathologic Characteristics and Differential Diagnosis. Mod. Pathol..

[27] Enzler T., Frankel T.L. (2025). Pancreatic Cancer Precursor Lesions—Can Immunotherapy Prevent Progression into Pancreatic Ductal Adenocarcinoma?. Cancer Lett..

[28] Buscail L., Bournet B., Cordelier P. (2020). Role of Oncogenic KRAS in the Diagnosis, Prognosis and Treatment of Pancreatic Cancer. Nat. Rev. Gastroenterol. Hepatol..

[29] Shendure J., Ji H. (2008). Next-Generation DNA Sequencing. Nat. Biotechnol..

[30] ICGC/TCGA Pan-Cancer Analysis of Whole Genomes Consortium (2020). Pan-Cancer Analysis of Whole Genomes. Nature.

[31] Bärthel S., Falcomatà C., Rad R., Theis F.J., Saur D. (2023). Single-Cell Profiling to Explore Pancreatic Cancer Heterogeneity, Plasticity and Response to Therapy. Nat. Cancer.

[32] Lichtenstein P., Holm N.V., Verkasalo P.K., Iliadou A., Kaprio J., Koskenvuo M., Pukkala E., Skytthe A., Hemminki K. (2000). Environmental and Heritable Factors in the Causation of Cancer—Analyses of Cohorts of Twins from Sweden, Denmark, and Finland. N. Engl. J. Med..

[33] Chen F., Childs E.J., Mocci E., Bracci P., Gallinger S., Li D., Neale R.E., Olson S.H., Scelo G., Bamlet W.R. (2019). Analysis of Heritability and Genetic Architecture of Pancreatic Cancer: A PanC4 Study. Cancer Epidemiol. Biomark. Prev..

[34] Roberts N.J., Norris A.L., Petersen G.M., Bondy M.L., Brand R., Gallinger S., Kurtz R.C., Olson S.H., Rustgi A.K., Schwartz A.G. (2016). Whole Genome Sequencing Defines the Genetic Heterogeneity of Familial Pancreatic Cancer. Cancer Discov..

[35] Abe T., Blackford A.L., Tamura K., Ford M., McCormick P., Chuidian M., Almario J.A., Borges M., Lennon A.M., Shin E.J. (2019). Deleterious Germline Mutations Are a Risk Factor for Neoplastic Progression Among High-Risk Individuals Undergoing Pancreatic Surveillance. J. Clin. Oncol..

[36] Overbeek K.A., Levink I.J.M., Koopmann B.D.M., Harinck F., Konings I.C.A.W., Ausems M.G.E.M., Wagner A., Fockens P., van Eijck C.H., Groot Koerkamp B. (2022). Long-Term Yield of Pancreatic Cancer Surveillance in High-Risk Individuals. Gut.

[37] Owens D.K., Davidson K.W., Krist A.H., Barry M.J., Cabana M., Caughey A.B., Curry S.J., Doubeni C.A., Epling J.W., US Preventive Services Task Force (2019). Screening for Pancreatic Cancer: US Preventive Services Task Force Reaffirmation Recommendation Statement. JAMA.

[38] Guidelines Detail. NCCN. https://www.nccn.org/guidelines/guidelines-detail?category=1&id=1455.

[39] Beger H.G., Mayer B., Poch B. (2024). Long-Term Oncologic Outcome Following Duodenum-Preserving Pancreatic Head Resection for Benign Tumors, Cystic Neoplasms, and Neuroendocrine Tumors: Systematic Review and Meta-Analysis. Ann. Surg. Oncol..

[40] Conroy T., Pfeiffer P., Vilgrain V., Lamarca A., Seufferlein T., O’Reilly E.M., Hackert T., Golan T., Prager G., Haustermans K. (2023). Pancreatic Cancer: ESMO Clinical Practice Guideline for Diagnosis, Treatment and Follow-Up. Ann. Oncol..

[41] Korrel M., Jones L.R., van Hilst J., Balzano G., Björnsson B., Boggi U., Bratlie S.O., Busch O.R., Butturini G., Capretti G. (2023). Minimally Invasive versus Open Distal Pancreatectomy for Resectable Pancreatic Cancer (DIPLOMA): An International Randomised Non-Inferiority Trial. Lancet Reg. Health Eur..

[42] Klotz R., Mihaljevic A.L., Kulu Y., Sander A., Klose C., Behnisch R., Joos M.C., Kalkum E., Nickel F., Knebel P. (2024). Robotic versus Open Partial Pancreatoduodenectomy (EUROPA): A Randomised Controlled Stage 2b Trial. Lancet Reg. Health Eur..

[43] Neoptolemos J.P., Stocken D.D., Friess H., Bassi C., Dunn J.A., Hickey H., Beger H., Fernandez-Cruz L., Dervenis C., Lacaine F. (2004). A Randomized Trial of Chemoradiotherapy and Chemotherapy after Resection of Pancreatic Cancer. N. Engl. J. Med..

[44] Oettle H., Post S., Neuhaus P., Gellert K., Langrehr J., Ridwelski K., Schramm H., Fahlke J., Zuelke C., Burkart C. (2007). Adjuvant Chemotherapy with Gemcitabine vs Observation in Patients Undergoing Curative-Intent Resection of Pancreatic Cancer: A Randomized Controlled Trial. JAMA.

[45] Neoptolemos J.P., Palmer D.H., Ghaneh P., Psarelli E.E., Valle J.W., Halloran C.M., Faluyi O., O’Reilly D.A., Cunningham D., Wadsley J. (2017). Comparison of Adjuvant Gemcitabine and Capecitabine with Gemcitabine Monotherapy in Patients with Resected Pancreatic Cancer (ESPAC-4): A Multicentre, Open-Label, Randomised, Phase 3 Trial. Lancet.

[46] Topalian S.L., Forde P.M., Emens L.A., Yarchoan M., Smith K.N., Pardoll D.M. (2023). Neoadjuvant Immune Checkpoint Blockade: A Window of Opportunity to Advance Cancer Immunotherapy. Cancer Cell.

[47] Stea E.X., Glantzounis G.K., Lykoudis E.G., Roukos D.H. (2026). Combination Therapy in Neoadjuvant and Metastatic Settings in Hot Tumors: Strengths and Weaknesses. Expert Rev. Anticancer Ther..

[48] Ghaneh P., Palmer D., Cicconi S., Jackson R., Halloran C.M., Rawcliffe C., Sripadam R., Mukherjee S., Soonawalla Z., Wadsley J. (2023). Immediate Surgery Compared with Short-Course Neoadjuvant Gemcitabine plus Capecitabine, FOLFIRINOX, or Chemoradiotherapy in Patients with Borderline Resectable Pancreatic Cancer (ESPAC5): A Four-Arm, Multicentre, Randomised, Phase 2 Trial. Lancet Gastroenterol. Hepatol..

[49] Katz M.H.G., Shi Q., Meyers J., Herman J.M., Chuong M., Wolpin B.M., Ahmad S., Marsh R., Schwartz L., Behr S. (2022). Efficacy of Preoperative mFOLFIRINOX vs mFOLFIRINOX Plus Hypofractionated Radiotherapy for Borderline Resectable Adenocarcinoma of the Pancreas. JAMA Oncol..

[50] Schwarz L., Bachet J.-B., Meurisse A., Bouché O., Assenat E., Piessen G., Hammel P., Regenet N., Taieb J., Turrini O. (2025). Neoadjuvant FOLF(IRIN)OX Chemotherapy for Resectable Pancreatic Adenocarcinoma: A Multicenter Randomized Noncomparative Phase II Trial (PANACHE01 FRENCH08 PRODIGE48 Study). J. Clin. Oncol..

[51] Labori K.J., Bratlie S.O., Andersson B., Angelsen J.-H., Biörserud C., Björnsson B., Bringeland E.A., Elander N., Garresori H., Grønbech J.E. (2024). Neoadjuvant FOLFIRINOX versus Upfront Surgery for Resectable Pancreatic Head Cancer (NORPACT-1): A Multicentre, Randomised, Phase 2 Trial. Lancet Gastroenterol. Hepatol..

[52] Yamaguchi J., Yokoyama Y., Fujii T., Yamada S., Takami H., Kawashima H., Ohno E., Ishikawa T., Maeda O., Ogawa H. (2022). Results of a Phase II Study on the Use of Neoadjuvant Chemotherapy (FOLFIRINOX or GEM/Nab-PTX) for Borderline-Resectable Pancreatic Cancer (NUPAT-01). Ann. Surg..

[53] Unno M., Motoi F., Matsuyama Y., Satoi S., Toyama H., Matsumoto I., Aosasa S., Shirakawa H., Wada K., Fujii T. (2026). Neoadjuvant Chemotherapy with Gemcitabine and S-1 Versus Upfront Surgery for Resectable Pancreatic Cancer: Results of the Randomized Phase II/III Prep-02/JSAP05 Trial. Ann. Surg..

[54] Sohal D.P.S., Duong M., Ahmad S.A., Gandhi N.S., Beg M.S., Wang-Gillam A., Wade J.L., Chiorean E.G., Guthrie K.A., Lowy A.M. (2021). Efficacy of Perioperative Chemotherapy for Resectable Pancreatic Adenocarcinoma: A Phase 2 Randomized Clinical Trial. JAMA Oncol..

[55] Katz M.H.G., Shi Q., Ahmad S.A., Herman J.M., Marsh R.d.W., Collisson E., Schwartz L., Frankel W., Martin R., Conway W. (2016). Preoperative Modified FOLFIRINOX Treatment Followed by Capecitabine-Based Chemoradiation for Borderline Resectable Pancreatic Cancer. JAMA Surg..

[56] Nagakawa Y., Hosokawa Y., Nakayama H., Sahara Y., Takishita C., Nakajima T., Hijikata Y., Kasuya K., Katsumata K., Tokuuye K. (2017). A Phase II Trial of Neoadjuvant Chemoradiotherapy with Intensity-Modulated Radiotherapy Combined with Gemcitabine and S-1 for Borderline-Resectable Pancreatic Cancer with Arterial Involvement. Cancer Chemother. Pharmacol..

[57] Parikh P.J., Lee P., Low D.A., Kim J., Mittauer K.E., Bassetti M.F., Glide-Hurst C.K., Raldow A.C., Yang Y., Portelance L. (2023). A Multi-Institutional Phase 2 Trial of Ablative 5-Fraction Stereotactic Magnetic Resonance-Guided On-Table Adaptive Radiation Therapy for Borderline Resectable and Locally Advanced Pancreatic Cancer. Int. J. Radiat. Oncol..

[58] Jeon H.J., Jeong H.J., Lim S.Y., Yoon S.J., Kim H., Han I.W., Heo J.S., Shin S.H. (2022). Pathological Response Predicts Survival after Pancreatectomy Following Neoadjuvant FOLFIRINOX for Pancreatic Cancer. Cancers.

[59] Tamburrino D., Arcangeli C., De Stefano F., Belfiori G., Macchini M., Orsi G., Schiavo Lena M., Partelli S., Crippa S., Doglioni C. (2024). Pathologic Complete Response Following Neoadjuvant Chemotherapy in Pancreatic Ductal Adenocarcinoma: Impact on Survival and Recurrence. Surgery.

[60] Seufferlein T., Uhl W., Kornmann M., Algül H., Friess H., König A., Ghadimi M., Gallmeier E., Bartsch D.K., Lutz M.P. (2023). Perioperative or Only Adjuvant Gemcitabine plus Nab-Paclitaxel for Resectable Pancreatic Cancer (NEONAX)-a Randomized Phase II Trial of the AIO Pancreatic Cancer Group. Ann. Oncol..

[61] Jang J.-Y., Han Y., Lee H., Kim S.-W., Kwon W., Lee K.-H., Oh D.-Y., Chie E.K., Lee J.M., Heo J.S. (2018). Oncological Benefits of Neoadjuvant Chemoradiation with Gemcitabine Versus Upfront Surgery in Patients with Borderline Resectable Pancreatic Cancer: A Prospective, Randomized, Open-Label, Multicenter Phase 2/3 Trial. Ann. Surg..

[62] Versteijne E., van Dam J.L., Suker M., Janssen Q.P., Groothuis K., Akkermans-Vogelaar J.M., Besselink M.G., Bonsing B.A., Buijsen J., Busch O.R. (2022). Neoadjuvant Chemoradiotherapy Versus Upfront Surgery for Resectable and Borderline Resectable Pancreatic Cancer: Long-Term Results of the Dutch Randomized PREOPANC Trial. J. Clin. Oncol..

[63] Cass S.H., Tzeng C.-W.D., Prakash L.R., Maxwell J., Snyder R.A., Kim M.P., Huey R.W., Smaglo B.G., Pant S., Koay E.J. (2025). Trends Over Time in Recurrence Patterns and Survival Outcomes after Neoadjuvant Therapy and Surgery for Pancreatic Cancer. Ann. Surg..

[64] Van Goor I.W.J.M., Schouten T.J., Verburg D.N., Besselink M.G., Bonsing B.A., Bosscha K., Brosens L.A.A., Busch O.R., Cirkel G.A., van Dam R.M. (2024). Predicting Long-Term Disease-Free Survival After Resection of Pancreatic Ductal Adenocarcinoma: A Nationwide Cohort Study. Ann. Surg..

[65] Blank C.U., Lucas M.W., Scolyer R.A., van de Wiel B.A., Menzies A.M., Lopez-Yurda M., Hoeijmakers L.L., Saw R.P.M., Lijnsvelt J.M., Maher N.G. (2024). Neoadjuvant Nivolumab and Ipilimumab in Resectable Stage III Melanoma. N. Engl. J. Med..

[66] Schmid P., Cortes J., Dent R., Pusztai L., McArthur H., Kümmel S., Bergh J., Denkert C., Park Y.H., Hui R. (2022). Event-Free Survival with Pembrolizumab in Early Triple-Negative Breast Cancer. N. Engl. J. Med..

[67] Tsuboi M., Herbst R.S., John T., Kato T., Majem M., Grohé C., Wang J., Goldman J.W., Lu S., Su W.-C. (2023). Overall Survival with Osimertinib in Resected EGFR-Mutated NSCLC. N. Engl. J. Med..

[68] Sorscher S. (2024). Perioperative Nivolumab in Resectable Lung Cancer. N. Engl. J. Med..

[69] Ceyhan Y., Garcia N.M.G., Alvarez J.V. (2023). Immune Cells in Residual Disease and Recurrence. Trends Cancer.

[70] Russo M., Chen M., Mariella E., Peng H., Rehman S.K., Sancho E., Sogari A., Toh T.S., Balaban N.Q., Batlle E. (2024). Cancer Drug-Tolerant Persister Cells: From Biological Questions to Clinical Opportunities. Nat. Rev. Cancer.

[71] Pantel K., Cote R.J., Fodstad O. (1999). Detection and Clinical Importance of Micrometastatic Disease. J. Natl. Cancer Inst..

[72] Sakamoto H., Attiyeh M.A., Gerold J.M., Makohon-Moore A.P., Hayashi A., Hong J., Kappagantula R., Zhang L., Melchor J.P., Reiter J.G. (2020). The Evolutionary Origins of Recurrent Pancreatic Cancer. Cancer Discov..

[73] Filis P., Kyrochristos I., Korakaki E., Baltagiannis E.G., Thanos D., Roukos D.H. (2023). Longitudinal ctDNA Profiling in Precision Oncology and Immunο-Oncology. Drug Discov. Today.

[74] Mordant P., Loriot Y., Lahon B., Castier Y., Lesèche G., Soria J.-C., Massard C., Deutsch E. (2012). Minimal Residual Disease in Solid Neoplasia: New Frontier or Red-Herring?. Cancer Treat. Rev..

[75] Wang X., Shen W., Yao L., Li C., You H., Guo D. (2025). Current Status and Future Prospects of Molecular Imaging in Targeting the Tumor Immune Microenvironment. Front. Immunol..

[76] Pantazopoulou V., Kubota C.S., Ogawa S., Gulay K.C.M., Lin X., Song H., Weitz J.R., Tiriac H., Lowy A.M., Engle D.D. (2025). Experimental Models of Pancreas Cancer: What Has Been the Impact for Precision Medicine?. J. Clin. Investig..

[77] Lencioni G., Gregori A., Toledo B., Rebelo R., Immordino B., Amrutkar M., Xavier C.P.R., Kocijančič A., Pandey D.P., Perán M. (2024). Unravelling the Complexities of Resistance Mechanism in Pancreatic Cancer: Insights from in Vitro and Ex-Vivo Model Systems. Semin. Cancer Biol..

[78] Hamburger A.W., Salmon S.E. (1977). Primary Bioassay of Human Tumor Stem Cells. Science.

[79] Ricci-Vitiani L., Lombardi D.G., Pilozzi E., Biffoni M., Todaro M., Peschle C., De Maria R. (2007). Identification and Expansion of Human Colon-Cancer-Initiating Cells. Nature.

[80] Vermeulen L., Sprick M.R., Kemper K., Stassi G., Medema J.P. (2008). Cancer Stem Cells—Old Concepts, New Insights. Cell Death Differ..

[81] Clevers H. (2011). The Cancer Stem Cell: Premises, Promises and Challenges. Nat. Med..

[82] De Sousa e Melo F., Kurtova A.V., Harnoss J.M., Kljavin N., Hoeck J.D., Hung J., Anderson J.E., Storm E.E., Modrusan Z., Koeppen H. (2017). A Distinct Role for Lgr5+ Stem Cells in Primary and Metastatic Colon Cancer. Nature.

[83] Fumagalli A., Oost K.C., Kester L., Morgner J., Bornes L., Bruens L., Spaargaren L., Azkanaz M., Schelfhorst T., Beerling E. (2020). Plasticity of Lgr5-Negative Cancer Cells Drives Metastasis in Colorectal Cancer. Cell Stem Cell.

[84] Heinz M.C., Peters N.A., Oost K.C., Lindeboom R.G.H., van Voorthuijsen L., Fumagalli A., van der Net M.C., de Medeiros G., Hageman J.H., Verlaan-Klink I. (2022). Liver Colonization by Colorectal Cancer Metastases Requires YAP-Controlled Plasticity at the Micrometastatic Stage. Cancer Res..

[85] Cañellas-Socias A., Cortina C., Hernando-Momblona X., Palomo-Ponce S., Mulholland E.J., Turon G., Mateo L., Conti S., Roman O., Sevillano M. (2022). Metastatic Recurrence in Colorectal Cancer Arises from Residual EMP1+ Cells. Nature.

[86] Chu X., Tian W., Ning J., Xiao G., Zhou Y., Wang Z., Zhai Z., Tanzhu G., Yang J., Zhou R. (2024). Cancer Stem Cells: Advances in Knowledge and Implications for Cancer Therapy. Signal Transduct. Target. Ther..

[87] Passaro A., Al Bakir M., Hamilton E.G., Diehn M., André F., Roy-Chowdhuri S., Mountzios G., Wistuba I.I., Swanton C., Peters S. (2024). Cancer Biomarkers: Emerging Trends and Clinical Implications for Personalized Treatment. Cell.

[88] Weinstein J.N., Collisson E.A., Mills G.B., Shaw K.R.M., Ozenberger B.A., Ellrott K., Shmulevich I., Sander C., Stuart J.M., Cancer Genome Atlas Research Network (2013). The Cancer Genome Atlas Pan-Cancer Analysis Project. Nat. Genet..

[89] Rahal Z., El Darzi R., Moghaddam S.J., Cascone T., Kadara H. (2025). Tumour and Microenvironment Crosstalk in NSCLC Progression and Response to Therapy. Nat. Rev. Clin. Oncol..

[90] Deveson I.W., Gong B., Lai K., LoCoco J.S., Richmond T.A., Schageman J., Zhang Z., Novoradovskaya N., Willey J.C., Jones W. (2021). Evaluating the Analytical Validity of Circulating Tumor DNA Sequencing Assays for Precision Oncology. Nat. Biotechnol..

[91] Jácome A.A., Johnson B. (2023). Minimal Residual Disease in Colorectal Cancer: Are We Finding the Needle in a Haystack?. Cells.

[92] Chaudhuri A.A., Chabon J.J., Lovejoy A.F., Newman A.M., Stehr H., Azad T.D., Khodadoust M.S., Esfahani M.S., Liu C.L., Zhou L. (2017). Early Detection of Molecular Residual Disease in Localized Lung Cancer by Circulating Tumor DNA Profiling. Cancer Discov..

[93] Corcoran R.B., Chabner B.A. (2018). Application of Cell-Free DNA Analysis to Cancer Treatment. N. Engl. J. Med..

[94] Parikh A.R., Van Seventer E.E., Siravegna G., Hartwig A.V., Jaimovich A., He Y., Kanter K., Fish M.G., Fosbenner K.D., Miao B. (2021). Minimal Residual Disease Detection Using a Plasma-Only Circulating Tumor DNA Assay in Patients with Colorectal Cancer. Clin. Cancer Res..

[95] Martínez-Castedo B., Camblor D.G., Martín-Arana J., Carbonell-Asins J.A., García-Micó B., Gambardella V., Huerta M., Roselló S., Roda D., Gimeno-Valiente F. (2025). Minimal Residual Disease in Colorectal Cancer. Tumor-Informed versus Tumor-Agnostic Approaches: Unraveling the Optimal Strategy. Ann. Oncol..

[96] Santonja A., Cooper W.N., Eldridge M.D., Edwards P.A.W., Morris J.A., Edwards A.R., Zhao H., Heider K., Couturier D.-L., Vijayaraghavan A. (2023). Comparison of Tumor-Informed and Tumor-Naïve Sequencing Assays for ctDNA Detection in Breast Cancer. EMBO Mol. Med..

[97] Kobayashi S., Nakamura Y., Hashimoto T., Bando H., Oki E., Karasaki T., Horinouchi H., Ozaki Y., Iwata H., Kato T. (2025). Japan Society of Clinical Oncology Position Paper on Appropriate Clinical Use of Molecular Residual Disease (MRD) Testing. Int. J. Clin. Oncol..

[98] Cecchini M., Salem R.R., Robert M., Czerniak S., Blaha O., Zelterman D., Rajaei M., Townsend J.P., Cai G., Chowdhury S. (2024). Perioperative Modified FOLFIRINOX for Resectable Pancreatic Cancer: A Nonrandomized Controlled Trial. JAMA Oncol..

[99] Du Z., Chen S., Qin Y., Lv Y., Du X., Yu H., Liu Z. (2025). Efficacy and Safety of Perioperative Immunotherapy for Patients with Non-Small Cell Lung Cancer: A Systematic Review and Network Meta-Analysis. Curr. Oncol..

[100] Agliano A., Calvo A., Box C. (2017). The Challenge of Targeting Cancer Stem Cells to Halt Metastasis. Semin. Cancer Biol..

[101] Cortina C., Turon G., Stork D., Hernando-Momblona X., Sevillano M., Aguilera M., Tosi S., Merlos-Suárez A., Stephan-Otto Attolini C., Sancho E. (2017). A Genome Editing Approach to Study Cancer Stem Cells in Human Tumors. EMBO Mol. Med..

[102] Batlle E., Clevers H. (2017). Cancer Stem Cells Revisited. Nat. Med..

[103] Takebe N., Miele L., Harris P.J., Jeong W., Bando H., Kahn M., Yang S.X., Ivy S.P. (2015). Targeting Notch, Hedgehog, and Wnt Pathways in Cancer Stem Cells: Clinical Update. Nat. Rev. Clin. Oncol..

[104] Clara J.A., Monge C., Yang Y., Takebe N. (2020). Targeting Signalling Pathways and the Immune Microenvironment of Cancer Stem Cells—A Clinical Update. Nat. Rev. Clin. Oncol..

[105] Majumder S., Crabtree J.S., Golde T.E., Minter L.M., Osborne B.A., Miele L. (2021). Targeting Notch in Oncology: The Path Forward. Nat. Rev. Drug Discov..

[106] Yang Y., Li X., Wang T., Guo Q., Xi T., Zheng L. (2020). Emerging Agents That Target Signaling Pathways in Cancer Stem Cells. J. Hematol. Oncol..

[107] Ryan M.B., Corcoran R.B. (2018). Therapeutic Strategies to Target RAS-Mutant Cancers. Nat. Rev. Clin. Oncol..

[108] Hallin J., Engstrom L.D., Hargis L., Calinisan A., Aranda R., Briere D.M., Sudhakar N., Bowcut V., Baer B.R., Ballard J.A. (2020). The KRASG12C Inhibitor MRTX849 Provides Insight toward Therapeutic Susceptibility of KRAS-Mutant Cancers in Mouse Models and Patients. Cancer Discov..

[109] Canon J., Rex K., Saiki A.Y., Mohr C., Cooke K., Bagal D., Gaida K., Holt T., Knutson C.G., Koppada N. (2019). The Clinical KRAS(G12C) Inhibitor AMG 510 Drives Anti-Tumour Immunity. Nature.

[110] Mahadevan K.K., McAndrews K.M., LeBleu V.S., Yang S., Lyu H., Li B., Sockwell A.M., Kirtley M.L., Morse S.J., Moreno Diaz B.A. (2023). KRASG12D Inhibition Reprograms the Microenvironment of Early and Advanced Pancreatic Cancer to Promote FAS-Mediated Killing by CD8+ T Cells. Cancer Cell.

[111] Stea E.X., Glantzounis G.K., Kydonakis N., Roukos D.H. (2025). Molecular Deciphering of Melanoma: Advances and Challenges of Neoadjuvant and Perioperative Treatment. Expert Rev. Mol. Diagn..

[112] Royal R.E., Levy C., Turner K., Mathur A., Hughes M., Kammula U.S., Sherry R.M., Topalian S.L., Yang J.C., Lowy I. (2010). Phase 2 Trial of Single Agent Ipilimumab (Anti-CTLA-4) for Locally Advanced or Metastatic Pancreatic Adenocarcinoma. J. Immunother..

[113] O’Reilly E.M., Oh D.-Y., Dhani N., Renouf D.J., Lee M.A., Sun W., Fisher G., Hezel A., Chang S.-C., Vlahovic G. (2019). Durvalumab with or Without Tremelimumab for Patients with Metastatic Pancreatic Ductal Adenocarcinoma: A Phase 2 Randomized Clinical Trial. JAMA Oncol..

[114] Yarchoan M., Johnson B.A., Lutz E.R., Laheru D.A., Jaffee E.M. (2017). Targeting Neoantigens to Augment Antitumour Immunity. Nat. Rev. Cancer.

[115] Marabelle A., Le D.T., Ascierto P.A., Di Giacomo A.M., De Jesus-Acosta A., Delord J.-P., Geva R., Gottfried M., Penel N., Hansen A.R. (2020). Efficacy of Pembrolizumab in Patients with Noncolorectal High Microsatellite Instability/Mismatch Repair-Deficient Cancer: Results From the Phase II KEYNOTE-158 Study. J. Clin. Oncol..

[116] Coston T., Desai A., Babiker H., Sonbol M.B., Chakrabarti S., Mahipal A., McWilliams R., Ma W.W., Bekaii-Saab T.S., Stauffer J. (2023). Efficacy of Immune Checkpoint Inhibition and Cytotoxic Chemotherapy in Mismatch Repair-Deficient and Microsatellite Instability-High Pancreatic Cancer: Mayo Clinic Experience. JCO Precis. Oncol..

[117] Liu Y.-T., Wang Y.-L., Wang S., Li J.-J., He W., Fan X.-J., Wan X.-B. (2025). Turning Cold Tumors into Hot Tumors to Ignite Immunotherapy. Mol. Cancer.

[118] Lopez de Rodas M., Villalba-Esparza M., Sanmamed M.F., Chen L., Rimm D.L., Schalper K.A. (2025). Biological and Clinical Significance of Tumour-Infiltrating Lymphocytes in the Era of Immunotherapy: A Multidimensional Approach. Nat. Rev. Clin. Oncol..

[119] Gaebler D., Hachey S.J., Hughes C.C.W. (2024). Microphysiological Systems as Models for Immunologically ‘Cold’ Tumors. Front. Cell Dev. Biol..

[120] Zaidi N., Jaffee E.M., Yarchoan M. (2025). Recent Advances in Therapeutic Cancer Vaccines. Nat. Rev. Cancer.

[121] Van der Leun A.M., Thommen D.S., Schumacher T.N. (2020). CD8+ T Cell States in Human Cancer: Insights from Single-Cell Analysis. Nat. Rev. Cancer.

[122] Zheng L., Qin S., Si W., Wang A., Xing B., Gao R., Ren X., Wang L., Wu X., Zhang J. (2021). Pan-Cancer Single-Cell Landscape of Tumor-Infiltrating T Cells. Science.

[123] Franklin M.R., Platero S., Saini K.S., Curigliano G., Anderson S. (2022). Immuno-Oncology Trends: Preclinical Models, Biomarkers, and Clinical Development. J. Immunother. Cancer.

[124] Thomas S.J., Moreira E.D., Kitchin N., Absalon J., Gurtman A., Lockhart S., Perez J.L., Pérez Marc G., Polack F.P., Zerbini C. (2021). Safety and Efficacy of the BNT162b2 mRNA COVID-19 Vaccine through 6 Months. N. Engl. J. Med..

[125] Balachandran V.P., Łuksza M., Zhao J.N., Makarov V., Moral J.A., Remark R., Herbst B., Askan G., Bhanot U., Senbabaoglu Y. (2017). Identification of Unique Neoantigen Qualities in Long-Term Survivors of Pancreatic Cancer. Nature.

[126] Łuksza M., Sethna Z.M., Rojas L.A., Lihm J., Bravi B., Elhanati Y., Soares K., Amisaki M., Dobrin A., Hoyos D. (2022). Neoantigen Quality Predicts Immunoediting in Survivors of Pancreatic Cancer. Nature.

[127] Bailey P., Chang D.K., Forget M.-A., Lucas F.A.S., Alvarez H.A., Haymaker C., Chattopadhyay C., Kim S.-H., Ekmekcioglu S., Grimm E.A. (2016). Exploiting the Neoantigen Landscape for Immunotherapy of Pancreatic Ductal Adenocarcinoma. Sci. Rep..

[128] Schumacher T.N., Schreiber R.D. (2015). Neoantigens in Cancer Immunotherapy. Science.

[129] Lander E.S., Linton L.M., Birren B., Nusbaum C., Zody M.C., Baldwin J., Devon K., Dewar K., Doyle M., FitzHugh W. (2001). Initial Sequencing and Analysis of the Human Genome. Nature.

[130] Venter J.C., Adams M.D., Myers E.W., Li P.W., Mural R.J., Sutton G.G., Smith H.O., Yandell M., Evans C.A., Holt R.A. (2001). The Sequence of the Human Genome. Science.

[131] Roukos D.H. (1999). Current Advances and Changes in Treatment Strategy May Improve Survival and Quality of Life in Patients with Potentially Curable Gastric Cancer. Ann. Surg. Oncol..

[132] Roukos D.H., Kappas A.M., Tsianos E. (2002). Role of Surgery in the Prophylaxis of Hereditary Cancer Syndromes. Ann. Surg. Oncol..

[133] Roukos D.H. (2010). Next-Generation, Genome Sequencing-Based Biomarkers: Concerns and Challenges for Medical Practice. Biomark. Med..

[134] Aparicio S., Caldas C. (2013). The Implications of Clonal Genome Evolution for Cancer Medicine. N. Engl. J. Med..

[135] Shendure J., Balasubramanian S., Church G.M., Gilbert W., Rogers J., Schloss J.A., Waterston R.H. (2017). DNA Sequencing at 40: Past, Present and Future. Nature.

[136] Roukos D.H. (2017). Spatiotemporal Diversification of Intrapatient Genomic Clones and Early Drug Development Concepts Realize the Roadmap of Precision Cancer Medicine. Drug Discov. Today.

[137] Wang K., Wang X., Pan Q., Zhao B. (2023). Liquid Biopsy Techniques and Pancreatic Cancer: Diagnosis, Monitoring, and Evaluation. Mol. Cancer.

[138] Dickey E.M., Martos M.P., Yanala U., Corona A., Ezenwajiaku N., Pizzolato J., Franceschi D., Livingstone A.S., Terrero G., Hester C.A. (2025). Utility of Tumor-Informed Circulating Tumor DNA for Detection of Minimal Residual Disease after Curative-Intent Therapy in Localized Pancreatic Cancer. Surg. Oncol. Insight.

[139] Lee B., Lipton L., Cohen J., Tie J., Javed A.A., Li L., Goldstein D., Burge M., Cooray P., Nagrial A. (2019). Circulating Tumor DNA as a Potential Marker of Adjuvant Chemotherapy Benefit Following Surgery for Localized Pancreatic Cancer. Ann. Oncol..

[140] Kitahata Y., Kawai M., Hirono S., Okada K.-I., Miyazawa M., Motobayashi H., Ueno M., Hayami S., Miyamoto A., Yamaue H. (2022). Circulating Tumor DNA as a Potential Prognostic Marker in Patients with Borderline-Resectable Pancreatic Cancer Undergoing Neoadjuvant Chemotherapy Followed by Pancreatectomy. Ann. Surg. Oncol..

[141] Botta G.P., Abdelrahim M., Drengler R.L., Aushev V.N., Esmail A., Laliotis G., Brewer C.M., George G.V., Abbate S.M., Chandana S.R. (2024). Association of Personalized and Tumor-Informed ctDNA with Patient Survival Outcomes in Pancreatic Adenocarcinoma. Oncologist.

[142] Hata T., Mizuma M., Motoi F., Ohtsuka H., Nakagawa K., Morikawa T., Unno M. (2023). Prognostic Impact of Postoperative Circulating Tumor DNA as a Molecular Minimal Residual Disease Marker in Patients with Pancreatic Cancer Undergoing Surgical Resection. J. Hepato-Biliary-Pancreat. Sci..

[143] Jiang J., Ye S., Xu Y., Chang L., Hu X., Ru G., Guo Y., Yi X., Yang L., Huang D. (2020). Circulating Tumor DNA as a Potential Marker to Detect Minimal Residual Disease and Predict Recurrence in Pancreatic Cancer. Front. Oncol..

[144] Yamaguchi T., Uemura K., Murakami Y., Kondo N., Nakagawa N., Okada K., Seo S., Hiyama E., Takahashi S., Sueda T. (2021). Clinical Implications of Pre- and Postoperative Circulating Tumor DNA in Patients with Resected Pancreatic Ductal Adenocarcinoma. Ann. Surg. Oncol..

[145] Lee J.-S., Han Y., Yun W.-G., Kwon W., Kim H., Jeong H., Seo M.-S., Park Y., Cho S.I., Kim H. (2022). Parallel Analysis of Pre- and Postoperative Circulating Tumor DNA and Matched Tumor Tissues in Resectable Pancreatic Ductal Adenocarcinoma: A Prospective Cohort Study. Clin. Chem..

[146] Lapin M., Tjensvoll K., Edland K.H., Oltedal S., Garresori H., Gilje B., Ekedal S., Eftestøl T., Kvaløy J.T., Janku F. (2025). Tumor-Agnostic Detection of Circulating Tumor DNA in Patients with Advanced Pancreatic Cancer Using Targeted DNA Methylation Sequencing and Cell-Free DNA Fragmentomics. Mol. Oncol..

[147] U.S. Food and Drug Administration (2026). List of Cleared or Approved Companion Diagnostic Devices (In Vitro and Imaging Tools).

[148] Volders P.-J., Aftimos P., Dedeurwaerdere F., Martens G., Canon J.-L., Beniuga G., Froyen G., Van Huysse J., De Pauw R., Prenen H. (2025). A Nationwide Comprehensive Genomic Profiling and Molecular Tumor Board Platform for Patients with Advanced Cancer. npj Precis. Oncol..

[149] Agritelley E.S., Ramaker R.C., Bao M., Bolch E., Allen P.J., Strickler J.H., Nussbaum D.P. (2025). Preparing for the Inevitable: Early Comprehensive Genomic Profiling for Patients with Localized Pancreatic Ductal Adenocarcinoma. JCO Oncol. Pract..

[150] Quail D.F., Joyce J.A. (2013). Microenvironmental Regulation of Tumor Progression and Metastasis. Nat. Med..

[151] Giraldo N.A., Sanchez-Salas R., Peske J.D., Vano Y., Becht E., Petitprez F., Validire P., Ingels A., Cathelineau X., Fridman W.H. (2019). The Clinical Role of the TME in Solid Cancer. Br. J. Cancer.

[152] Roukos D.H. (2008). Innovative Genomic-Based Model for Personalized Treatment of Gastric Cancer: Integrating Current Standards and New Technologies. Expert Rev. Mol. Diagn..

[153] Pan D., Li X., Qiao X., Wang Q. (2025). Immunosuppressive Tumor Microenvironment in Pancreatic Cancer: Mechanisms and Therapeutic Targets. Front. Immunol..

[154] Ma K., Wang L., Li W., Tang T., Ma B., Zhang L., Zhang L. (2025). Turning Cold into Hot: Emerging Strategies to Fire up the Tumor Microenvironment. Trends Cancer.

[155] Schmitt M.W., Loeb L.A., Salk J.J. (2016). The Influence of Subclonal Resistance Mutations on Targeted Cancer Therapy. Nat. Rev. Clin. Oncol..

[156] Mo C.-K., Liu J., Chen S., Storrs E., Targino da Costa A.L.N., Houston A., Wendl M.C., Jayasinghe R.G., Iglesia M.D., Ma C. (2024). Tumour Evolution and Microenvironment Interactions in 2D and 3D Space. Nature.

[157] Chen K., Chen Z., Wang J., Zhou M., Liu Y., Xu B., Yu Z., Li Y., Yang G., Xu T. (2025). Single-Cell Sequencing Unravels Pancreatic Cancer: Novel Technologies Reveal Novel Aspects of Cellular Heterogeneity and Inform Therapeutic Strategies. Biomedicines.

[158] Kyrochristos I.D., Roukos D.H. (2019). Comprehensive Intra-Individual Genomic and Transcriptional Heterogeneity: Evidence-Based Colorectal Cancer Precision Medicine. Cancer Treat. Rev..

[159] Roukos D.H. (2014). Genome Network Medicine: Innovation to Overcome Huge Challenges in Cancer Therapy. Wiley Interdiscip. Rev. Syst. Biol. Med..

[160] Gohil S.H., Iorgulescu J.B., Braun D.A., Keskin D.B., Livak K.J. (2021). Applying High-Dimensional Single-Cell Technologies to the Analysis of Cancer Immunotherapy. Nat. Rev. Clin. Oncol..

[161] Guo T., Steen J.A., Mann M. (2025). Mass-Spectrometry-Based Proteomics: From Single Cells to Clinical Applications. Nature.

[162] (2021). Method of the Year 2020: Spatially Resolved Transcriptomics. Nat. Methods.

[163] (2024). Method of the Year 2024: Spatial Proteomics. Nat. Methods.

[164] Meric-Bernstam F., Larkin J., Tabernero J., Bonini C. (2021). Enhancing Anti-Tumour Efficacy with Immunotherapy Combinations. Lancet.

[165] Plana D., Palmer A.C., Sorger P.K. (2022). Independent Drug Action in Combination Therapy: Implications for Precision Oncology. Cancer Discov..

[166] Jin H., Wang L., Bernards R. (2023). Rational Combinations of Targeted Cancer Therapies: Background, Advances and Challenges. Nat. Rev. Drug Discov..

[167] Hartupee C., Nagalo B.M., Chabu C.Y., Tesfay M.Z., Coleman-Barnett J., West J.T., Moaven O. (2024). Pancreatic Cancer Tumor Microenvironment Is a Major Therapeutic Barrier and Target. Front. Immunol..

[168] Sethna Z., Guasp P., Reiche C., Milighetti M., Ceglia N., Patterson E., Lihm J., Payne G., Lyudovyk O., Rojas L.A. (2025). RNA Neoantigen Vaccines Prime Long-Lived CD8+ T Cells in Pancreatic Cancer. Nature.

[169] Qiao Y., Yin H., Zhang Y., Zhang J., Dong Q. (2025). Domestication and Feedback: Bidirectional Hijacking in Pancreatic Ductal Adenocarcinoma Microenvironment. Front. Immunol..

[170] Quail D.F., Walsh L.A. (2024). Revolutionizing Cancer Research with Spatial Proteomics and Visual Intelligence. Nat. Methods.

[171] Shakiba M., Tuveson D.A. (2025). Macrophages and Fibroblasts as Regulators of the Immune Response in Pancreatic Cancer. Nat. Immunol..

[172] Oliveira G., Wu C.J. (2023). Dynamics and Specificities of T Cells in Cancer Immunotherapy. Nat. Rev. Cancer.

[173] Gong D., Arbesfeld-Qiu J.M., Perrault E., Bae J.W., Hwang W.L. (2024). Spatial Oncology: Translating Contextual Biology to the Clinic. Cancer Cell.

[174] Du L., Yang H. (2025). Spatial Omics in 3D Culture Model Systems: Decoding Cellular Positioning Mechanisms and Microenvironmental Dynamics. J. Transl. Med..

[175] Pentimalli T.M., Schallenberg S., León-Periñán D., Legnini I., Theurillat I., Thomas G., Boltengagen A., Fritzsche S., Nimo J., Ruff L. (2025). Combining Spatial Transcriptomics and ECM Imaging in 3D for Mapping Cellular Interactions in the Tumor Microenvironment. Cell Syst..

[176] Larson C.R., Mandloi A., Acharyya S., Carstens J.L. (2025). The Tumor Microenvironment across Four Dimensions: Assessing Space and Time in Cancer Biology. Front. Immunol..

[177] Pantel K., Andersen C.L., Schuuring E., Malats N., Heitzer E., Vat L., Kiernan E., Chin E., Barrett J.C., Kapp J.R. (2023). 237TiP GUIDE.MRD: A Consortium Guiding Multi-Modal Therapies against Minimal Residual Disease (MRD) by Liquid Biopsy to Assess Implementation of Circulating Tumor DNA (ctDNA) in Clinical Practice to Improve Patient Outcomes. Ann. Oncol..

